# Field evaluation of maize genotypes for resistance to pre-harvest *Aspergillus* ear rot and aflatoxin contamination under natural inoculation in Eastern and Northern Tanzania

**DOI:** 10.3389/fpls.2026.1834989

**Published:** 2026-06-05

**Authors:** Mbwana Macheli Abdallah, Richard Raphael Madege, Luseko Amos Chilagane

**Affiliations:** 1 Department of Crop Science and Horticulture, Sokoine University of Agriculture, Morogoro, Tanzania; 2 Selian Centre, Tanzania Agricultural Research Institute, Arusha, Tanzania

**Keywords:** aflatoxin, *Aspergillus* ear rot, *Aspergillus flavus*, maize genotypes, natural infestation, pre-harvest

## Abstract

Maize, a staple for billions of people, is highly vulnerable to aflatoxin contamination caused by *Aspergillus flavus*. Aflatoxins pose serious threats and risks to food safety, public health, and trade. This study aimed to evaluate and identify superior maize genotypes as potential sources of resistance for sustainable breeding programmes focused on high yield and aflatoxin resistance, ultimately reducing aflatoxin exposure. Twelve (12) genotypes were evaluated under natural aflatoxin-prone conditions using a 4 × 3 alpha lattice design across three locations: Ilonga-Kilosa, SUA-Morogoro, and Kirusix-Babati. Phenological, agronomic, and yield-related data were collected, and harvested ears were visually scored for *Aspergillus* ear rot (AER). Aflatoxin levels in kernels were analysed using Ultra High-Performance Liquid Chromatography (UHPLC). Genotype (G) and environment (E) significantly influenced ear rot (ER), grain moisture (GM), and field weight (FW) (p < 0.05). G × E interactions were significant for ER and GM but not FW. Grain yield ranged from 1.63 to 5.54 t/ha (mean: 3.59 t/ha), with SUA recording the highest yield and lowest aflatoxin levels. Aflatoxin contamination varied from 0.16 to 14.34 µg/kg (mean: 4.225 µg/kg), with 50% of samples exceeding the EU safety limit of 4 µg/kg. Correlations between aflatoxin levels and agronomic traits/parameters related to aflatoxin contamination differed across environments, highlighting strong genotype and environmental effects. Principal Component Analysis (PCA) identified plant height, ear height, and flowering time as key contributors to aflatoxin contamination. These findings emphasise the need for breeding high-yielding, AER-resistant genotypes with reduced aflatoxin levels to enhance maize productivity and food safety.

## 1 Introduction

Agriculture remains the primary means of survival worldwide, providing food for humans and animals alike. Food security is achieved when all individuals consistently have physical and economic access to sufficient, safe, and nutritious food, supporting an active and healthy life (Pérez-Escamilla, 2017). Maize (*Zea mays* L.) is one of the world’s leading crops, thriving in tropics, subtropics, and temperate regions (Solaimalai et al., 2020). In many parts of Africa, maize has become the favourite cereal for food, feed, and industrial use.

However, maize production is increasingly threatened by aflatoxin contamination, which results from pre- and post-harvest infection by toxigenic fungi, particularly *Aspergillus flavus* and *Aspergillus parasiticus* (Frisvad et al., 2019; Logrieco et al., 2018; Mahuku et al., 2019). Aflatoxins (AFB1, AFB2, AFG1, and AFG2), especially AFB1, are highly toxic and carcinogenic, yet their presence often is ignored because they do not directly affect crop yield (Udomkun et al., 2017; Shabeer et al., 2022). Their production is influenced by insect damage, drought and heat stress, and other environmental and management factors, with significant spatial and temporal variability even within localities (Boni et al., 2021; Magembe et al., 2016; Mannaa and Kim, 2017; Richard, 2007; White, 2016).

Globally, aflatoxin contamination remains a major food safety challenge, posing serious health risks to both humans and livestock (Benkerroum, 2020). The FAO estimates that approximately 25% of food crops are contaminated with mycotoxins, while more recent studies indicate a higher prevalence of 60–80% when both food and feed are considered (Eskola et al., 2020; Kebede et al., 2020; Shabeer et al., 2022). Empirical evidence further confirms widespread contamination, with 72–79% of feed samples testing positive in large-scale surveys (Kovalsky et al., 2016; Streit et al., 2013). Regional cereal assessments suggest that contamination burdens are generally higher in Africa and Asia compared to Europe and the Americas (Niaz et al., 2025). Consequently, more than 4.5 billion people are estimated to be at risk of chronic aflatoxicosis (Joutsjoki and Korhonen, 2021; Ponce-García et al., 2021), with documented outbreaks and fatalities in Tanzania emphasizing its current public health burden (Kamala et al., 2018).

Aflatoxin contamination not only poses serious health risks but also results in substantial economic losses due to the rejection of contaminated grain in both domestic and international markets (Udomkun et al., 2017). Trade restrictions, including temporary bans on maize imports within the East African region, further highlight its economic impact (Food Business MEA, 2021; The Citizen, 2021). To safeguard consumers and ensure food safety, regulatory limits have been established: the U.S. Food and Drug Administration allows up to 20 μg/kg of total aflatoxins in maize (Cai et al., 2020), while stricter limits apply in East Africa at 10 μg/kg (East African Community, 2011; Massomo, 2020) and in the European Union at 4 μg/kg (European Commission, 2010).

Previous studies on aflatoxin mitigation have focused more on management practices and biological control. However, these approaches are often limited by cost, environmental variability, and inconsistent field performance (Abdallah et al., 2018). A key challenge is that aflatoxin accumulation is a low-heritability trait strongly influenced by genotype × environment (G × E) interactions, leading to unstable outcomes across environments (Odindo, 2017; Okoth et al., 2009).

Although maize landraces represent a rich source of genetic diversity and high adaptability, especially in diverse agro-ecologies such as Tanzania, few studies have systematically evaluated their potential for aflatoxin resistance. Most research has instead focused on improved germplasm, leaving locally adapted landraces underutilized despite their potential for stable performance under variable conditions (Caldu-Primo et al., 2017; Dwivedi et al., 2016; Romero Navarro et al., 2017; Warburton et al., 2017). This study addresses this by evaluating maize landraces, to identify stable sources of reduced aflatoxin accumulation for breeding programs.

## 2 Materials and methods

### 2.1 Description of research area

#### 2.1.1 Field experiment

**Figure 1:**
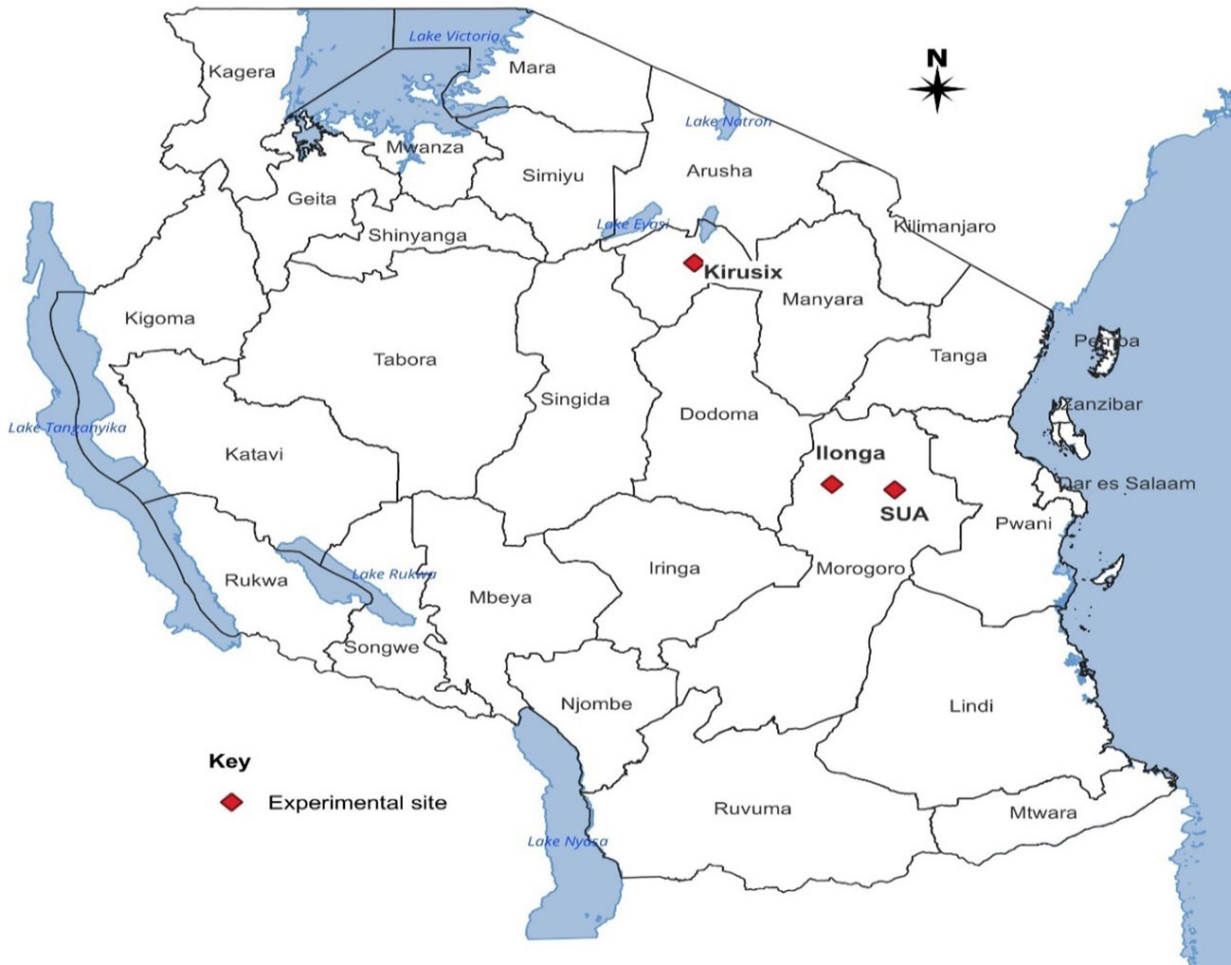
Map of Tanzania showing three sites where trials were conducted. Source: Open Map Sources (OMS), National Bureau of Statistics (NBS) and experimental sites.

The trials were conducted across three locations in Eastern and Northern Tanzania to capture diverse agro-ecological conditions (**Figure 1**). The first site was Ilonga-Kilosa, located at a latitude of -6.834, a longitude of 36.991, and an altitude of 491 metres above sea level (a.s.l.). The second site was the Sokoine University of Agriculture (SUA) Crop Museum in Morogoro, positioned at a latitude of -6.850, a longitude of 37.649, and an altitude of 526 metres a.s.l. The third site, Kirusix-Babati, was situated at a latitude of -4.038, a longitude of 35.828, and an altitude of 1158 meters a.s.l. These sites were selected based on previous research on aflatoxin contamination as documented by Boni et al. (2021); Kamala et al. (2016); Magembe et al. (2016), and Nyangi (2016). The weather data (Tanzania Meteorological Authority, 2024), including rainfall, temperature, and humidity, for the study sites were recorded and are presented in **Table 1**.

**Table 1:** Monthly mean temperature, rainfall, and relative humidity at Kilosa, Morogoro, and Babati in 2023.

Months (2023)	Temperature (°C)			Rainfall (mm)			Relative humidity (%)		
	Ilonga	SUA	Kirusix	Ilonga	SUA	Kirusix	Ilonga	SUA	Kirusix
January	22.4	23.8	20.2	151.8	140.5	37.8	85.3	82.6	71.0
February	22.9	24.5	21.7	113.6	248.1	49.4	82.9	79.4	66.6
March	23.6	25.0	21.6	111.1	120.3	76.5	78.4	76.7	68.5
April	22.4	23.5	20.4	176.4	300.0	137.7	86.3	86.3	76.5
May	21.0	22.0	19.7	89.0	189.5	56.0	86.5	87.2	75.1
June	19.8	20.7	18.7	78.7	262.1	27.8	82.4	83.4	74.0
July	20.0	20.9	18.2	13.1	9.5	1.1	72.6	75.4	67.8
August	21.7	22.8	17.8	32.8	33.3	238.0	69.2	71.0	76.2
September	24.1	25.1	20.2	19.1	48.8	8.0	62.5	63.3	69.9
October	25.7	26.7	21.3	53.1	51.1	40.8	61.4	63.0	65.7
November	23.2	24.5	20.0	287.7	348.5	370.0	83.2	83.3	81.6
December	23.7	24.9	20.6	158.3	155.5	89.7	85.7	85.0	81.2

Source: Tanzania Meteorological Agency (TMA).

### 2.2 Plant materials

Twelve maize genotypes were selected from 64 (58 landraces and 6 checks) based on genetic divergence from cluster analysis using morphological and molecular data. The selected set included 9 landraces and 3 improved varieties. For maturity classification, genotypes 1788 and A2207-4 (108 days) were classified as medium-maturing, while the remaining genotypes (>110 days) were considered late-maturing (Bello et al., 2012; Oluwarant et al., 2015). The list of genotypes evaluated for *Aspergillus* ear rot (AER) and aflatoxin resistance is presented in **Table 2**.

**Table 2:** Maize genotypes used in trial for AER and aflatoxin resistance screening.

SN	Maize genotype	Type of genotype	Maturity time	Status/objective	Source of maize genotype
1	1	Landraces	Medium	Test genotype	TPGRC (Tanzania)
2	2743	Landraces	Medium	Test genotype	TPGRC (Tanzania)
3	1769	Landraces	Late	Test genotype	TPGRC (Tanzania)
4	A2207-4	Improved	Late	Check	IITA (Nigeria)
5	A2207-1	Improved	Late	Check	IITA (Nigeria)
6	5110	Landraces	Late	Test genotype	TPGRC (Tanzania)
7	2438	Landraces	Late	Test genotype	TPGRC (Tanzania)
8	AFL-Syn2-y	Improved	Late	Check	IITA (Nigeria)
9	1788	Landraces	Late	Test genotype	TPGRC (Tanzania)
10	YANGA-5-2	Landraces	Late	Test genotype	TPGRC (Tanzania)
11	12	Landraces	Late	Test genotype	TPGRC (Tanzania)
12	2246	Landraces	Late	Test genotype	TPGRC (Tanzania)

TPGRC, Tanzania Plant Genetic Resources Centre; IITA, International Institute for Tropical Agriculture.

### 2.3 Field evaluation of maize genotypes against *Aspergillus* ear rot and aflatoxin contamination

#### 2.3.1 Establishment layout and trial establishment

Twelve maize genotypes were evaluated using a 4 × 3 alpha lattice design with three replicates across three sites. Each plot consisted of two 3 m rows planted at 75 cm × 30 cm spacing, with one plant per hill after thinning. Standard agronomic practices were applied, including two weeding at 4 and 7 weeks after emergence following pre-emergence glyphosate application. Fertilizer was applied as 10 kg P/ha at planting and 80 kg N/ha as urea in two splits at 4 and 7 weeks after emergence. Planting was done on January 1, 2023 (Ilonga), January 13, 2023 (SUA), and April 2, 2023 (Kirusix), with dates adjusted according to rainfall onset and site-specific agro-ecological conditions.

#### 2.3.2 Data collection

The number of seedlings after emergence was counted, and days to anthesis and silking were recorded as the number of days from planting to when 50% of plants shed pollen and emerged silks, respectively. The anthesis-silking interval (ASI) was calculated as the difference between days to 50% silking and 50% anthesis. Plant and ear heights were measured in centimetres, from the base to the first tassel branch and from the base to the node bearing the upper ear, respectively.

Foliar disease severity, including rust, blight, grey leaf spot (GLS), maize lethal necrosis (MLN), and maize streak virus (MSV). The methodology has been strengthened by clearly defining both the scoring system and the symptom-based criteria used for disease identification. Specifically, each foliar disease was diagnosed based on its characteristic field symptoms prior to scoring. Foliar disease severity (rust, blight, grey leaf spot, MLN, and MSV) was assessed using a 1–9 scale, where 1 indicated no symptoms and 9 indicated severe infection. Each disease was first identified based on characteristic field symptoms (e.g., pustules for rust, grey lesions for GLS, chlorotic streaks for MSV, and severe chlorosis and necrosis for MLN). Standardized guidelines to ensure consistency and improve data reliability across all sites. Disease scoring was conducted at the 10th week after emergence.

Plant and ear aspects were scored on a 1–5 scale, where 1 represented excellent phenotypic appeal and clean, well-filled ears, while 5 indicated poor plant appeal and small, rotten ears. Kernel texture was scored on scale of 1-5, where 1 very flint and 5 very dents. All harvested ears were weighed, and grain moisture content was determined using representative samples. Grain yield, adjusted to 12.5% moisture, was computed from ear weight and grain moisture levels. *Aspergillus* ear rot was assessed based on visual symptoms, including powdery yellow-green or olive mold on kernels and cobs. Other recorded data were lodging (stem and root), plant and ear count at harvest and husk cover.

These data were important for evaluating maize genotypes for aflatoxin resistance because they provided information on adaptation, plant health, ear protection, and grain susceptibility under field conditions. Traits such as flowering time, anthesis–silking interval, foliar disease severity, grain moisture, husk cover, and *Aspergillus* ear rot helped identify factors influencing fungal infection and aflatoxin accumulation, thereby enabling the identification of genotypes with stable performance and lower risk of contamination across environments.

### 2.4 Quantification of aflatoxins in tested maize genotypes

Maize grain samples were collected from experimental sites at Ilonga (Kilosa), SUA (Morogoro), and Kirusix (Babati) and sent to the International Livestock Research Institute (ILRI) laboratory in Nairobi, Kenya for fungal quantification.

#### 2.4.1 Maize grain sampling and sample preparation

Sampling was a critical step in aflatoxin analysis due to the uneven (skewed) distribution of aflatoxins in grain, which can lead to high sampling errors. To minimize this, grain sampling followed the Standard Operating Procedure (SOP) for Aflatoxin Testing (Version 1.1, 2010) and guidelines outlined by the Food and Agriculture Organization of the United Nations (2018) and by Herrman et al. (2014). After sampling, grains were milled using the Romer Series II Mill under standardized conditions to ensure sample homogeneity and reduce cross-contamination. Each sample was milled separately, with the mill thoroughly cleaned and flushed with blank grain material between samples. The milling process was conducted at a constant speed according to manufacturer recommendations to ensure uniform particle size. These procedures improved sample representativeness and enhanced the accuracy and reliability of aflatoxin detection for both research and regulatory purposes.

To assess the level of contamination in maize samples from which Aspergillus had been isolated, a subset of the maize was analysed for aflatoxin contamination. Twenty-five millilitres of 70% methanol was added into the 50-mL Falcon tube (BD, Franklin Lakes, NJ, USA) containing 5 g of milled maize. The mixture was vortexed for 1 min and shaken in a mechanical orbital shaker (New Brunswick, NJ, USA) at 350 rpm for one hour at room temperature. The extract was further centrifuged at 3500 rpm for 10 min. A total of 0.5 mL of the supernatant was transferred using a micropipette into clean 2-mL centrifuge tube, diluted further with 0.5 mL of 1% acetic acid and centrifuged at 13,000 rpm for 5 min. A volume of 0.7 mL of the diluent was transferred into HPLC vial for UPLC analysis.

#### 2.4.2 Extraction of aflatoxins from the maize samples

The procedure for extraction was described in AOAC Method No. 2005.08 (AOAC, 2006) and followed Doose et al. (2019) and Bilal et al. (2023) as follows; Maize samples were milled to approximately 0.5 mm particle size using a Romer Series II mill (Romer Labs, Getzersdorf, Austria). A 5 g subsample was accurately weighed into a 50 mL Falcon tube (BD, Franklin Lakes, NJ, USA), and 25 mL of 70% methanol was added. The mixture was vortexed for 1 min and shaken at 350 rpm for 1 hour at room temperature (21–25 °C) using an orbital shaker (New Brunswick, NJ, USA). It was then centrifuged at 3,500 rpm for 10 min under controlled temperature conditions to ensure proper phase separation and analyte stability. A 0.5 mL aliquot of the supernatant was transferred into a 2 mL tube, diluted with 0.5 mL of 1% acetic acid, and centrifuged again at 13,000 rpm for 5 min. Finally, 0.7 mL of the clarified extract was transferred into an HPLC vial for UPLC analysis.

#### 2.4.3 Analysis of aflatoxins using ultra-high-performance liquid chromatography

The aflatoxins B1, B2, G1, and G2 were quantified in accordance with the procedure described by AOAC Method No. 2005.08 (AOAC, 2006), Benvenuti and Gioia (2009), Doose et al. (2019) and later by Bilal et al. (2023) by using ultra-high-performance liquid chromatography-fluorescence detection (UHPLC-FLD) method as follows: Chromatographic separation was performed using a Nexera Ultra High-Performance Liquid Chromatography (UHPLC) system (Shimadzu Corporation, Kyoto, Japan) fitted with an auto sampler (SIL-30AC), prominence pumps (LC-20AD), and a fluorescence detector (RF-20AXS). A Synergi Hydro-RP (Phenomenex contains a reversed-phase C18 silica-based stationary phase with enhanced hydrophilic properties, enabling effective separation of aflatoxins under high aqueous mobile phase condition) analytical column (2.5 µm particle size, 100 mm x 3.00 mm) (Phenomenex, Torrance, CA, USA) operating at a flow rate of 0.4 mL/min was used for the separation of aflatoxins. A binary mobile phase, consisting of mobile phase A (methanol (40%)) and mobile phase B (1% acetic acid (60%)), was utilised to achieve this separation. The injection volume was 10 µL, and the column oven temperature was set at 50 °C. The liquid chromatography program was set at 8 min per run, and 60% methanol was used as the flushing solution of the column. Fluorescence detection was carried out at wavelengths of λex = 365 nm and λem = 435 nm. A standard calibration curve, consisting of a plot of peak areas against known concentrations of injected aflatoxin standards, was established for aflatoxins B1, B2, G1, and G2. The standards were obtained from a certified reference supplier, Sigma-Aldrich (Merck, Darmstadt, Germany), and were of analytical grade/certified reference material (CRM) grade with a stated purity of ≥98%. The calibration curve was then used to quantify aflatoxin concentrations in samples using LabSolutions software version 5.89 (Shimadzu Corporation, Kyoto, Japan, 2004). Individual types of aflatoxin were identified by comparing the retention time of the chromatographic peak of the target aflatoxin in the test sample to that of the corresponding standard chromatographic peak. Samples with values above the linear range of the standard curve were diluted and retested. The limit of detection (LOD) for aflatoxins B1, B2, G1, and G2 during the time of analysis was 0.360, 0.086, 0.223, and 0.072 µg/kg, respectively. LOD represents the smallest amount of aflatoxin that can be reliably detected, but not necessarily quantified with high accuracy. The concentration of individual aflatoxins in the test samples was calculated as follows:


$$X(\frac{ng}{g})=\frac{C\;x\;V\;x\;F\;x100}{W\;x\;R}$$

Where;

X—The total content of individual aflatoxin in the test sample, ng/g

C—The concentration of aflatoxin in the test sample, ng/mL, after calibration using LabSolutions software.

V – Extraction volume used in mL

F—Dilution factor after extraction with 1% acetic acid

100%—Percentage for recovery

W—The weight used of the test sample, g

R - Experimentally determined recovery factor from spike recovery experiment

Then, Total aflatoxin was estimated as the sum of individual aflatoxin types in each sample. Quality control included certified reference maize materials and blank solvents (70% methanol:1% acetic acid, 1:1). The reference materials were obtained from the Office of the Texas State Chemist Aflatoxin Proficiency Testing Program (APTECA), Chiromo Campus, Nairobi, Kenya. Results were considered accurate when values of reference materials fell within ±3 SD of the control chart for each analytical run (Doose et al., 2019).

#### 2.4.4 Statistical analysis

Phenological, agronomic, and yield-related data were analysed by ANOVA using GenStat 16th Edition at a 5% probability level, with mean separation by LSD and Tukey HSD at P ≤ 0.05. Aflatoxin concentrations were log-transformed as ln (y + 1) before analysis using the generalized linear model procedure in SAS 9.4, with significance set at P ≤ 0.05. Pearson correlation analysis was used to examine relationships between morphological traits and aflatoxin accumulation, while principal component analysis was performed in R 4.4.2 software (R Core Team, 2024) to identify traits contributing most to *Aspergillus* ear rot and aflatoxin contamination across sites.

## 3 Results

### 3.1 Assessment of various maize traits, variables and yield performance

#### 3.1.1 Assessment of agronomical, morphological and phenological traits in tested maize genotypes across the environments

Harvesting was conducted on May 26, May 28, and August 5, 2023, for Ilonga, SUA, and Kirusix, respectively. Kirusix recorded the highest plant aspect (3.00) and ear aspect (3.24) and was the latest flowering (FM = 65.47, FF = 68.78), indicating good plant quality, strong lodging resistance, and late maturity. SUA exhibited the tallest plants (PH = 193.47) with the highest ear height (EH = 110.83), while Ilonga showed the highest lodging (3.36), indicating poor standability; Kirusix had the lowest lodging (1.56), reflecting better stability (**Table 3**).

**Table 3:** Means of the main effects for the agronomical/morphological/ phenological traits in tested environments.

Sites	Agronomical/morphological/phenological traits								
	FM	FF	HC	Lodg	EH	PH	Text	PA	EA
SUA	59.03 b	61.61 b	1.94 ab	2.56 ab	110.83 a	193.47 a	2.11 a	2.44 b	2.08 b
Kirusix	65.47 a	68.78 a	2.13 a	1.56 b	90.28 b	164.00 b	2.01 a	3.00 a	3.24 a
Ilonga	60.11 b	62.83 b	1.90 b	3.36 a	88.47 b	167.08 b	2.00 a	3.22 a	3.21 a

Means with different letters differ significantly and vice versa (P ≤ 0.05) (Tukey's HSD); FM, Male flowering; FF, Female flowering; HC, Husk cover; Lodg, Lodging; EH, ear height; PH, Plant height; Text, kernel texture; PA, Plant aspect; EA, Ear aspect.

**Table 4** shows that genotype 5110 was the tallest (PH = 200.56, EH = 116.11), while AFL-Syn2-y was the shortest (PH = 155.56, EH = 82.22) and latest flowering (FM = 66.44, FF = 69.33). 1788 flowered earliest (FM = 58.22, FF = 61.67), 2743 had the highest lodging (3.00), and A2207–1 the lowest (1.67). 2246 had dent kernel texture (2.56), and A2207–4 had a flinty texture (1.61). The results show strong genetic variation among genotypes, indicating useful traits and trade-offs for selection in breeding programs.

**Table 4:** Means of the main effects for the agronomical/morphological/ phenological traits in tested genotypes.

Genotypes	Agronomical/morphological/phenological traits								
	HC	Lodg	EH	PH	Text	PA	EA	FM	FF
1	2.11 ab	2.33 a	113.89 ab	199.44 ab	1.83 bcd	3.11 ab	3.06 ab	62.56 bcd	65.22 bcd
2438	1.89 abc	2.44 a	114.44 a	192.78 abc	2.17 abc	3.28 ab	3.44 ab	62.78 bcd	65.78 abc
1788	2.28 ab	1.89 a	87.22 cd	163.89 d	1.94 bcd	3.44 a	3.72 a	58.22 e	61.67 de
A2207-4	1.83 abc	2.11 a	92.22 bcd	173.89 cd	1.61 d	2.22 c	2.67 b	58.11 e	60.78 e
12	2.17 ab	2.22 a	85.00 d	160.00 d	2.22 ab	3.78 abc	3.33 ab	60.11 cde	62.78 cde
2743	1.78 bc	3.00 a	89.44 cd	169.89 cd	2.00 bcd	3.22 ab	3.33 ab	59.89 de	62.44 cde
A2207-1	2.33 a	1.67 a	107.22 abc	177.78 abcd	1.722 cd	2.89 abc	3.06 ab	59.89 de	62.67 cde
AFL-Syn2-y	2.06 ab	3.44 a	82.22 d	155.56 d	1.89 bcd	3.11 ab	3.06 ab	66.44 a	69.33 a
YANGA-5-2	2.00 abc	2.78 a	87.78 cd	166.67 d	2.06 bcd	2.56 bc	2.67 b	58.56 e	61.56 de
1769	2.11 ab	2.89 a	85.00 d	162.78 d	2.22 ab	2.67 bc	3.17 ab	63.56 ab	66.67 ab
2246	1.83 abc	2.44 a	97.78 abcd	175.00 bcd	2.56 a	2.72 abc	2.67 b	62.56 bcd	65.22 bcd
5110	1.50 c	2.67 a	116.11 a	200.56 a	2.28 ab	2.67 bc	2.83 b	65.78 ab	68.78 ab

Means sharing the same letter are not significantly different and vice versa (Tukey's HSD).

ANOVA (**Table 5**) showed Environment (E) significantly affects EH, PH, and EA (p < 0.001), while Genotype (G) influences EH, PH, Text, and EA (p < 0.001). Significant G × E interaction (p < 0.05) highlights trait variability across environments, emphasising the need for multi-environment trials to select stable, resilient genotypes. E

**Table 5:** ANOVA table of agronomical/morphological/phenological traits in tested maize genotype (G), environment (E), and their interaction (G × E).

Source of variation	d.f	Mean Squares (M.S)					
		HC (F *Pr*)	Lodg (F *Pr*)	EH (F *Pr*)	PH (F *Pr*)	Text (F *Pr*)	EA (F *Pr*)
Environment (E)	2	0.5023 (0.0153) *	29.454 (0.0182) *	5555 (4.09e-10) ***	9447 (2.1e-12) ***	0.1319 (0.2639) ns	2.0903 (0.000543) ***
Genotype (G)	11	0.4992 (4.89e-05) ***	2.231 (0.9789) ns	1492 (7.21e-09) ***	2084 (1.1e-09) ***	0.6269 (2.47e-07) ***	1.0278 (0.000108) ***
G x E	22	0.1968 (0.0424) *	9.04 (0.2018) ns	318 (0.0492) *	531 (0.00546) **	0.2229 (0.0045) **	0.5574 (0.005822) **

F *Pr*, F-probability; Gx E, genotype by Environment Interaction.

#### 3.1.2 Assessment of foliar diseases in tested maize genotypes across the environments

Foliar disease severity varied by site, with Kirusix showing higher MLN (3.36) and MSV (1.53), and SUA recording the highest rust severity (2.03). GLS and Turc differed little across locations (**Table 6**). Overall, disease patterns were largely similar, except for MLN and rust, highlighting the importance of location effects and genotype × environment interactions and the need for multi-location testing.

**Table 6:** Means of the main effects for the foliar diseases in tested environments.

Sites	Foliar diseases				
	MLN	MSV	Rust	GLS	Turc
SUA	1.00a	1.00a	2.03c	1.17b	1.94b
Kirusix	3.36c	1.53b	1.39b	1.11ab	2.00b
Ilonga	1.00a	1.00a	1.17b	1.08a	1.61b

Means followed by the same letter within a column are not significantly different at P ≤ 0.05.

MLN, Maize Lethal Necrosis; MSV, Maize Streak Virus; GLS, Grey Leaf Spot; Turc, Turcicum Leaf Blight.

The results (**Table 7**) showed no significant differences in disease severity among genotypes for MLN, MSV, Rust, GLS, and Turc (P > 0.05). Meaning that genotypes had similar disease responses, with no real differences in resistance or susceptibility.

**Table 7:** Means of the main effects for the foliar diseases in tested genotypes.

Genotypes	Foliar diseases				
	MLN	MSV	Rust	GLS	Turc
1	1.89a	1.11a	1.78a	1.00a	2.11a
2438	1.89a	1.44a	1.78a	1.33a	2.11a
1788	1.67a	1.11a	1.67a	1.11a	2.56a
A2207-4	1.78a	1.44a	1.67a	1.00a	1.56a
12	1.78a	1.00a	1.56a	1.11a	1.78a
2743	1.67a	1.11a	1.56a	1.00a	2.11a
A2207-1	1.78a	1.11a	1.44a	1.11a	1.67a
AFL-Syn2-y	1.67a	1.00a	1.44a	1.00a	1.89a
YANGA-5-2	1.78a	1.22a	1.44a	1.11a	1.44a
1769	1.78a	1.22a	1.33a	1.22a	1.33a
2246	1.78a	1.11a	1.33a	1.11a	1.67a
5110	2.00a	1.22a	1.33a	1.33a	2.00a

Environmental conditions significantly influenced MLN, MSV, and Rust, while GLS and Turc remained stable across locations (**Table 8**). The absence of genotype and G × E effects indicates that all genotypes performed similarly, with no differences in resistance or specific adaptation. Overall, this suggests limited genetic variation, and that disease severity was mainly driven by environmental factors rather than genotype.

**Table 8:** ANOVA table of foliar diseases in tested maize genotype (G), environment (E), and their interaction (G × E).

Source of variation	d.f	Mean Squares (M.S)				
		MLN (F Pr)	MSV (F Pr)	Rust (F Pr)	GLS (F Pr)	Turc (F Pr)
Environment (E)	2	66.90 (2e-16) ***	3.343 (2.37e-07) ***	7.194 (4.56e-06) ***	0.06481(0.4999) ns	1.5926(0.118) ns
Genotype (G)	11	0.09 (0.739) ns	0.191 (0.385) ns	0.245 (0.898) ns	0.13047(0.1876) ns	1.0774(0.154) ns
G x E	22	0.09 (0.830) ns	0.191 (0.382) ns	0.477 (0.507) ns	0.14562(0.0782) ns	0.8451(0.301) ns

d.f, degree of freedom; F *Pr*, F-probability; G x E Genotype by environment interaction.

ns, Not significant, ***, significant.

#### 3.1.3 Assessment of *Aspergillus* ear rot and yield-related variables in tested maize genotypes

The results (**Table 9**) show that Genotype 1788 had the highest ear rot severity (9.3%), indicating higher fungal susceptibility, while 2743 and A2207–1 had the lowest (4.3%). 2246 had the highest fresh weight (2.53 kg), implying high yielding, and 5110 and A2207–4 had the highest grain moisture. A2207–4 also had the highest number of ears (19.67) and plants (18.56). These variations suggest that genotype plays a significant role in resistance to ear rot and overall productivity, which is crucial for breeding programmes focused on improving yield and reducing fungal contamination.

**Table 9:** Mean performance of maize genotypes for ear rot (ER) and yield-related traits (mean ± S.E.).

Factors and levels	Measured parameters				
(Genotypes)	ER (%)	FW (Kg)	GM (%)	NE	NP
1	6.1 ± 1.10 b	1.97 ± 0.25 bc	12.96 ± 0.58 ab	15.11 ± 1.73 ab	16.56 ± 1.25 ab
12	6.7 ± 1.10 b	1.93 ± 0.25 bc	12.99 ± 0.58 ab	16.33 ± 1.73 ab	17.33 ± 1.25 ab
1769	7.3 ± 1.10 bc	1.87 ± 0.25 bc	13.01 ± 0.58 ab	16.00 ± 1.73 ab	16.56 ± 1.25 ab
1788	9.3 ± 1.10 c	1.45 ± 0.25 a	11.67 ± 0.58 a	14.78 ± 1.73 ab	16.22 ± 1.25 ab
2246	4.9 ± 1.10 a	2.53 ± 0.25 d	13.46 ± 0.58 bc	15.56 ± 1.73 ab	16.56 ± 1.25 ab
2438	6.4 ± 1.10 b	1.76 ± 0.25 bc	11.87 ± 0.58 a	15.11 ± 1.73 ab	17.89 ± 1.25 ab
2743	4.3 ± 1.10 a	1.58 ± 0.25 ab	12.48 ± 0.58 ab	13.11 ± 1.73 a	14.00 ± 1.25 a
5110	5.3 ± 1.10 ab	2.20 ± 0.25 cd	13.67 ± 0.58 c	15.67 ± 1.73 ab	17.22 ± 1.25 ab
A2207-1	4.3 ± 1.10 a	1.71 ± 0.25 bc	13.30 ± 0.58 bc	16.33 ± 1.73 ab	14.78 ± 1.25 a
A2207-4	6.9 ± 1.10 bc	2.41 ± 0.25 d	13.77 ± 0.58 c	19.67 ± 1.73 b	18.56 ± 1.25 b
AFL-Syn2-y	5.3 ± 1.10 ab	2.06 ± 0.25 bc	13.11 ± 0.58 ab	15.44 ± 1.73 ab	16.00 ± 1.25 ab
YANGA-5-2	6.2 ± 1.10 b	2.19 ± 0.25 cd	13.03 ± 0.58 ab	16.22 ± 1.73 ab	18.00 ± 1.25 b

S.E, Standard Error; GM, Grain Moisture; FW, Field Weight; ER, Ear Rot, NP and NE, No. of Plants and Ears; Means of same letters are the same and vice versa.

#### 3.1.4 Assessment of AER and yield-related variables in tested environments

The results in **Table 10** show that Kirusix had the highest ear rot (6.78%), while SUA had the highest fresh weight (2.48 kg), grain moisture (14.36%), and number of ears (20.06). Ilonga had the lowest fresh weight (1.48 kg) and number of ears (14.08) but the highest number of plants (15.86). These findings highlight site-specific differences in fungal susceptibility and productivity, emphasising the role of environmental factors in improving yield and resistance.

**Table 10:** Means of the main effects for ear rot (ER) and yield-related variables in tested environments.

Factors and levels	Measured parameters				
(Sites)	ER (%)	FW (Kg)	GM (%)	NE	NP
Ilonga	4.92a	1.48a	12.77b	14.08b	15.86a
Kirusix	6.78b	1.96b	11.69a	13.19a	16.56a
SUA	6.61b	2.48c	14.36c	20.06c	17.5a

ER, Ear rot; FW, Field weight; GM, Grain moisture; NE and NP, No. of ears and plants.

#### 3.1.5 Assessment of AER and yield-related variables in maize genotypes and their interaction to environments

Genotype × environment (G × E) interactions significantly influenced maize traits across sites. SUA had the highest field weight (FW) (3.52 kg for 2246) and the most severe ear rot (ER) (14% for 1788), likely due to high temperature and humidity. Grain moisture (GM) varied, being higher at SUA and Ilonga than Kirusix (**Table 11**). Differences in the number of ears and plants highlighted genetic and environmental influences on plant establishment.

**Table 11:** Means of main effects on ER and yield-related variables for genotype and environment interaction (G x E).

Factors and levels		Measured parameters				
Site/environment	Genotype	FW (Kg)	ER (%)	GM (%)	NE	NP
Ilonga	2246	2.06abc	4bc	14.63a-e	15.33abc	17.67a
Ilonga	A2207-4	1.977abc	5bc	14.03a-f	17abc	16.33a
Ilonga	5110	1.913abc	5bc	14.03a-f	16abc	16.67a
Ilonga	AFL-Syn2-y	1.537bc	4.67bc	13.8a-f	14.33bc	15.33a
Ilonga	YANGA-5-2	1.487bc	4.33bc	12.97a-f	14bc	16.67a
Ilonga	2743	1.39bc	4.33bc	11.93a-f	12.33bc	13.33a
Ilonga	2438	1.36bc	5.67bc	10.17f	14.33bc	17a
Ilonga	A2207-1	1.36bc	2c	12.03a-f	16abc	17a
Ilonga	1	1.323bc	5bc	12.4a-f	12.67bc	14.33a
Ilonga	1769	1.123c	6bc	13.7a-f	14bc	16a
Ilonga	1788	1.107c	6.67abc	11.57a-f	10.67c	14.67a
Ilonga	12	1.1c	6.33bc	12a-f	12.33bc	15.33a
Kirusix	YANGA-5-2	2.287abc	8.33abc	11.27a-f	14.33bc	17.33a
Kirusix	A2207-4	2.203abc	6bc	12.33a-f	15bc	19.33a
Kirusix	5110	2.193abc	7.33abc	12.33a-f	12.67bc	16.67a
Kirusix	1	2.087abc	5bc	12.6a-f	14.33bc	16.67a
Kirusix	12	2.043abc	4.67bc	12.73a-f	14bc	17.33a
Kirusix	AFL-Syn2-y	2.043abc	5.33bc	10.67ef	11bc	14.67a
Kirusix	1788	2.04abc	7.33abc	10.83c-f	14.67bc	16a
Kirusix	2246	2.023abc	5.67bc	10.93b-f	11.33bc	16.33a
Kirusix	2438	1.697bc	10.33ab	11.13a-f	12.33bc	18.67a
Kirusix	1769	1.65bc	8.67abc	10.7ef	12.67bc	16.33a
Kirusix	2743	1.627bc	5.33bc	10.77d-f	11.67bc	14.67a
Kirusix	A2207-1	1.623bc	7.33abc	14.03a-f	14.33bc	14.67a
SUA	2246	3.52a	5bc	14.8a-c	20abc	15.67a
SUA	A2207-4	3.04ab	9.67ab	14.93a	27a	20a
SUA	1769	2.84abc	7.33abc	14.63a-e	21.33abc	17.33a
SUA	YANGA-5-2	2.803abc	6bc	14.87ab	20.33abc	20a
SUA	12	2.64abc	9ab	14.23a-e	22.67ab	19.33a
SUA	AFL-Syn2-y	2.597abc	6bc	14.87ab	21abc	18a
SUA	1	2.51abc	8.33abc	13.87a-f	18.33abc	18.67a
SUA	5110	2.493abc	3.67bc	14.63a-e	18.33abc	18.33a
SUA	2438	2.21abc	3.33bc	14.3a-e	18.67abc	18a
SUA	A2207-1	2.14abc	3.67bc	13.83a-f	18.67abc	12.67a
SUA	2743	1.733bc	3.33bc	14.73a-d	15.33abc	14a
SUA	1788	1.203c	14	12.6a-f	19abc	18a
S.E.D.		0.44	1.89	0.99	2.98	2.16

Means followed by the same letters are not significantly different (P < 0.05).

The ANOVA (**Table 12**) confirmed significant G × E effects on ER and GM but not FW. Overall, this suggests that environmental conditions strongly affect ER and GM in combination with genotype, while FW is more stable and less influenced by environmental variation or interaction effects.

**Table 12:** ANOVA table for ear rot, field weight, and grain moisture in tested maize genotypes across environments.

Source of variation	d.f	Mean squares (M.S)		
		Ear rot (*F pr.)*	Field weight (*F pr.)*	Grain moisture (*F pr.)*
Replication	2	4.45	1.07	4.03
Environment (E)	2	38.18 (0.03) *	8.99 (0.007) **	64.64 (<0.001) ***
Genotype (G)	11	17.95 (0) ***	0.95 (< 0.001) ***	3.80 (0.011) *
G X E	22	13.58 (0) ***	0.33 (0.269) ns	3.11 (0.014) *

*, **, *** significant at 0.05, 0.01, and 0.001 probability levels, respectively; d.f., degree of freedom; F pr., probability value; ns, not statistically significant.

#### 3.1.6 Yield performance of tested maize genotypes across the environments

**Table 13** shows varying maize yields across three sites, with SUA yielding the highest average (3.91 t/ha), followed by Kirusix (3.20 t/ha) and Ilonga (2.37 t/ha). Genotype 2246 had the highest yield across all sites, while genotype 1788 had the lowest. The results indicate significant yield variation in Ilonga, but not in SUA or Kirusix. These findings suggest that breeding programmes should focus on site-specific strategies, as local conditions affect maize performance differently across environments.

**Table 13:** Yield performance of tested genotypes across environments.

Genotypes	Grain yield (t/ha)			Average yield (across sites) (t/ha)	Rank (#)
	SUA	Ilonga	Kirusix		
1	3.99	1.94	3.37	3.10	6
1769	4.48	1.87	2.73	3.03	7
2246	5.54	3.13	3.32	4.00	1
2743	2.72	2.33	2.66	2.57	11
2438	3.50	2.28	2.80	2.86	9
YANGA-5-2	4.41	2.46	3.77	3.55	3
12	4.17	1.63	3.29	3.03	8
5110	3.93	3.09	3.52	3.51	4
A2207-4	4.77	3.17	3.57	3.84	2
A2207-1	3.41	2.34	2.56	2.77	10
1788	1.94	1.72	3.35	2.34	12
AFL-Syn2-y	4.08	2.50	3.42	3.33	5
Mean	3.91	2.37	3.20		
LSD (0.05)	2.12	0.87	0.93		
CV	30.70	20.72	12.58		
*p*	0.145	0.027	0.126		
*p *	ns	*	ns		
Min	1.94	1.63	2.56		
Max	5.54	3.17	3.77		

t/ha, tonnes per hectare; LSD, least significant difference; CV, coefficient of variation, p, probability value, *, means significant at p = 0.05, ns, not (non) significant; Min, Minimum; Max, maximum.

The two-way ANOVA (**Table 14**) presents yield result was significantly affected by environment, while genotype and G × E interaction were not significant, indicating that environmental conditions were the primary driver of yield variation across sites.

**Table 14:** ANOVA table assessing yield performance in tested maize genotype (G), environment (E), and their interaction (G × E).

Source of variation	df	SS	MS	F-value	p-value	Significance
Genotype (G)	11	4.62	0.42	1.43	0.145	ns
Environment (E)	2	6.20	3.10	5.32	0.027	*
G x E	22	5.34	0.24	1.29	0.126	ns
Error	73	42.56	0.58	–	–	–
Total	107	58.72	–	–	–	–

df, degree of freedom; SS/MM, sum/mean square; ns, non-significant; *significant; Gx E, GEI.

### 3.2 Pre-harvest aflatoxins quantification in harvested maize grains

#### 3.2.1 Effects of genotypes on pre-harvest aflatoxin contamination of maize

Aflatoxin contamination was highly significant among maize genotypes (p < 2e-16) (**Table 15**), with A2207–4 having the lowest level (1.33 µg/kg) and YANGA-5–2 the highest (7.65 µg/kg), as shown in **Table 16**. Genotypes A2207-4, 1, and AFL-Syn2-y showed higher resistance, while YANGA-5–2 and Genotype 12 exhibited greater susceptibility.

**Table 15:** ANOVA table for total aflatoxin on tested genotypes across environments.

Source of variation	d.f	SS	MS	F value	F pr
Environment (E)	2	118.8	59.41	1.13E+30	<2e-16 ***
Genotype (G)	11	421.7	38.34	7.26E+29	<2e-16 ***
G x E	22	1149.9	52.27	9.90E+29	<2e-16 ***

d.f, degree of freedom; SS, Sum squares; Mean squares; F *pr*, F probability.

**Table 16:** Aflatoxin accumulation levels in maize grains (genotype) at harvest.

Tested genotypes	TAF (µg/kg) across sites			Overall TAF per genotype(µg/kg)	Mean TAF per genotype (µg/kg)	Rank (#)
	Ilonga	SUA	Kirusix			
1	0.45	1.06	3.69	5.20	1.73	2
1769	5.00	1.53	1.64	8.17	2.72	4
2246	7.59	0.16	3.10	10.85	3.62	5
2743	7.83	2.68	3.23	13.73	4.58	8
2438	3.29	1.58	14.34	19.21	6.40	10
YANGA-5-2	12.19	6.22	4.55	22.96	7.65	11
12	15.12	0.63	4.37	20.12	6.71	12
5110	1.86	14.37	1.76	17.99	6.00	9
A2207-4	0.84	1.13	2.04	4.00	1.33	1
A2207-1	4.83	2.16	4.73	11.72	3.91	6
1788	5.15	2.47	4.67	12.29	4.10	7
AFL-Syn2-y	1.34	0.83	4.77	6.94	2.31	3
Total/site (µg/kg)	65.48	34.81	52.89			
Mean/site(µg/kg)	5.46	2.90	4.41			

TAF, total aflatoxin; µg/kg, microgram per kilogram; Site, Environment.

#### 3.2.2 Effects of environment on pre-harvest aflatoxin contamination of maize

Aflatoxin levels varied across sites and were highly significant (p < 2e-16), as shown in **Table 3**.16, with Ilonga (Kilosa) showing the highest contamination (5.46 µg/kg), followed by Kirusix (Babati) at 4.41 µg/kg and SUA (Morogoro) at 2.90 µg/kg (**Table 16**). Higher contamination in Ilonga is linked to environmental factors like higher humidity and low rains, especially from flowering to harvesting (**Table 1**).

#### 3.2.3 Effects of genotype by environment interaction on pre-harvest aflatoxin contamination of maize

**Figure 2:**
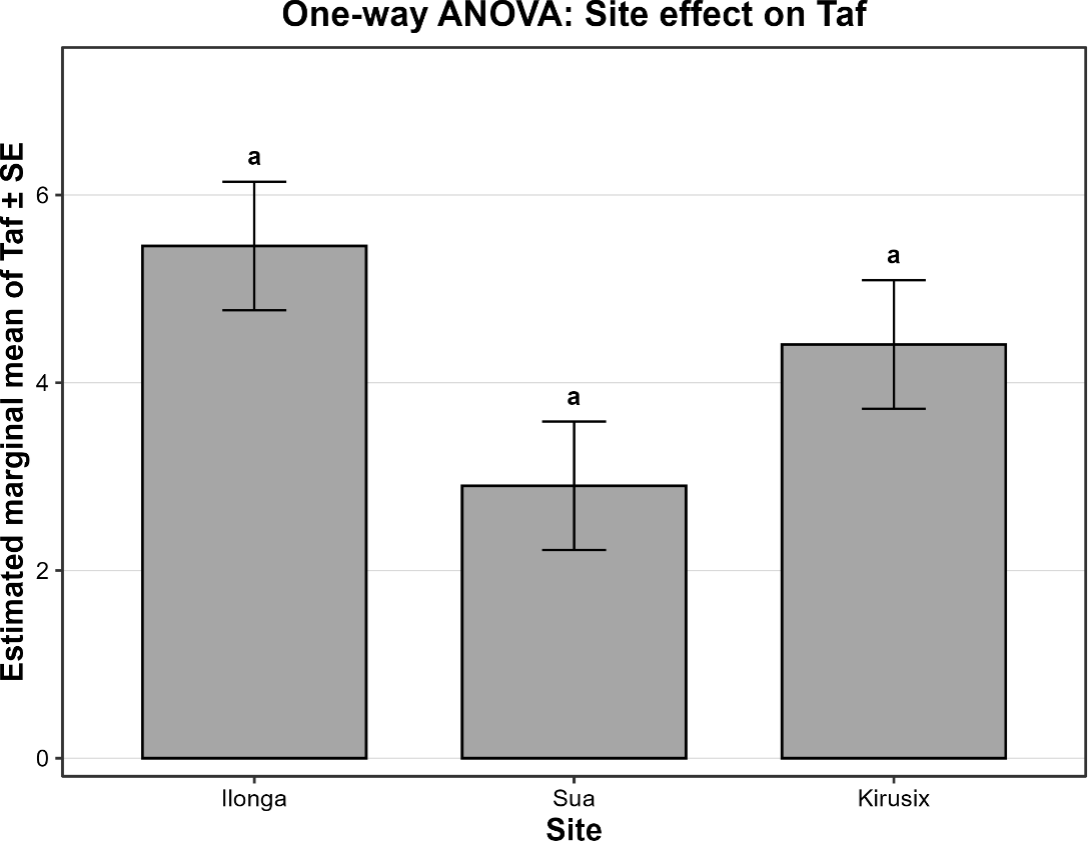
Effect of site on total aflatoxin (Taf) contents.

**Figure 3:**
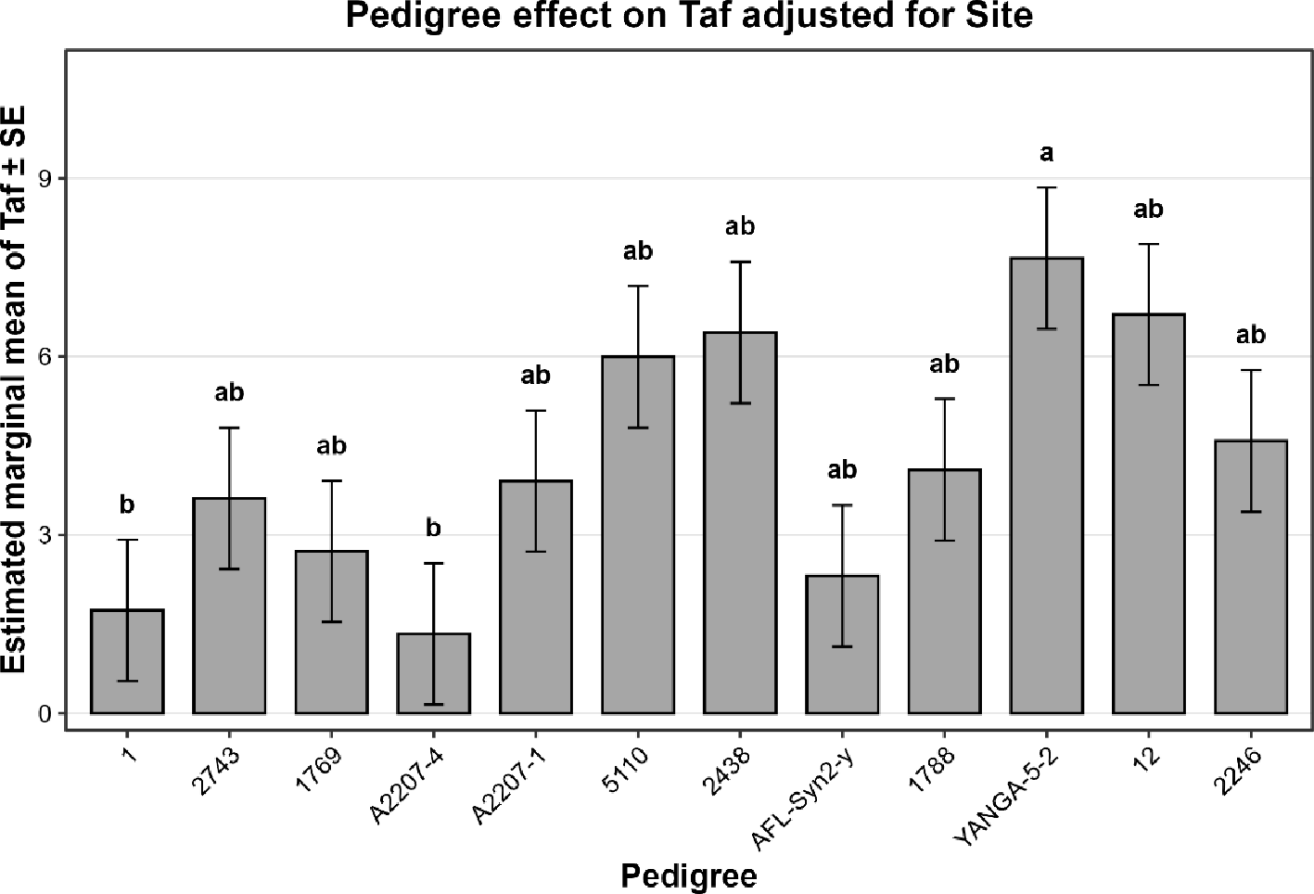
Effect of maize genotypes on total aflatoxin content adjusted for site.

**Figure 4:**
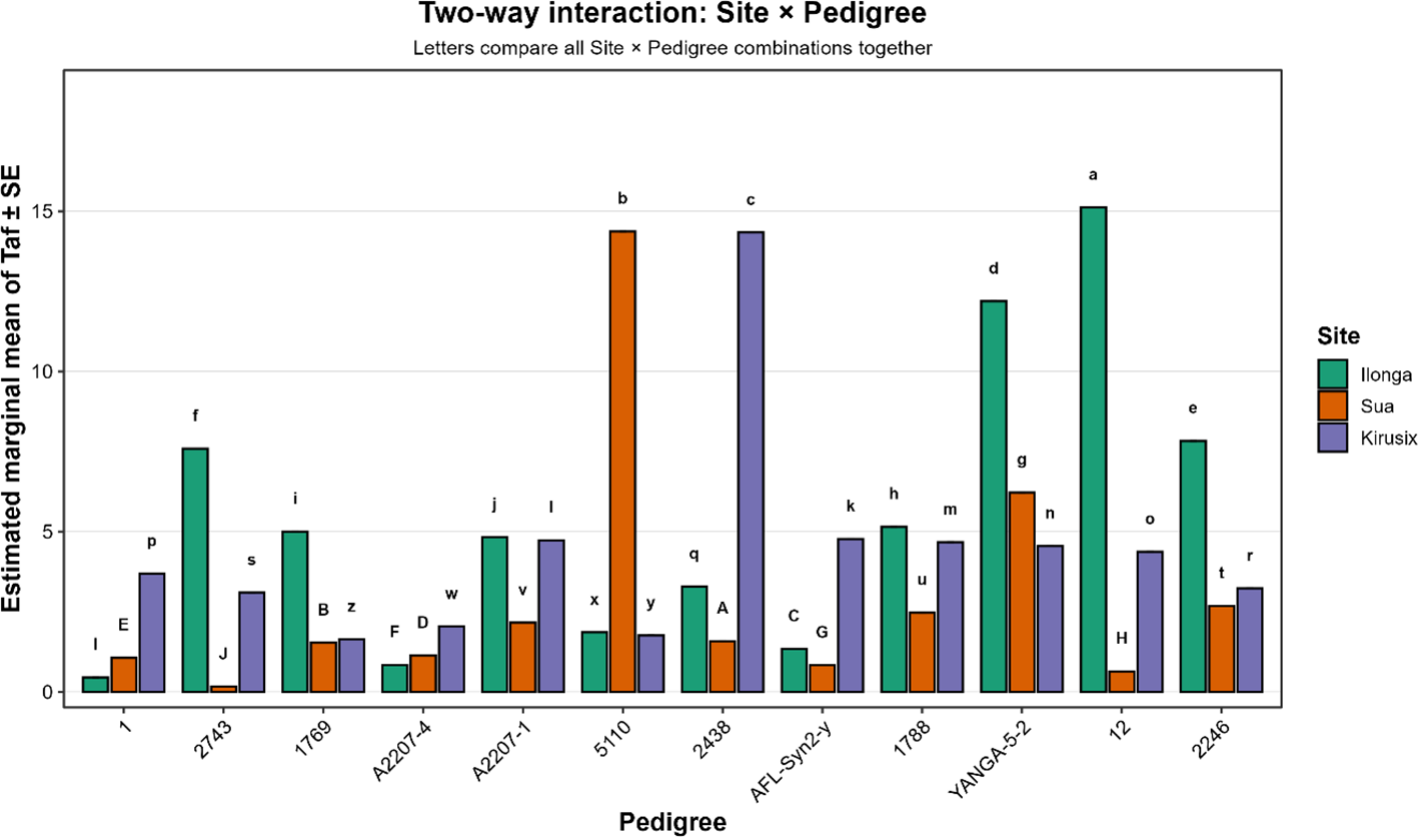
Interaction effects of sites and maize genotypes on total aflatoxin content.

The genotype × environment (G×E) interaction significantly influenced aflatoxin contamination (**Table 16**), with the highest contamination recorded at Ilonga (**Figure 2**). Some genotypes, including 12, 5110, and 2438, showed high susceptibility, whereas AFL-Syn2-y, 1, and A2207–4 consistently showed low contamination (**Figures 3**, **4**), suggesting genetic resistance. **Table 15** further confirmed highly significant effects of both G and E.

### 3.3 A pooled correlation matrix between morphological traits/parameters associated with aflatoxin and aflatoxin contamination

**Figure 5:**
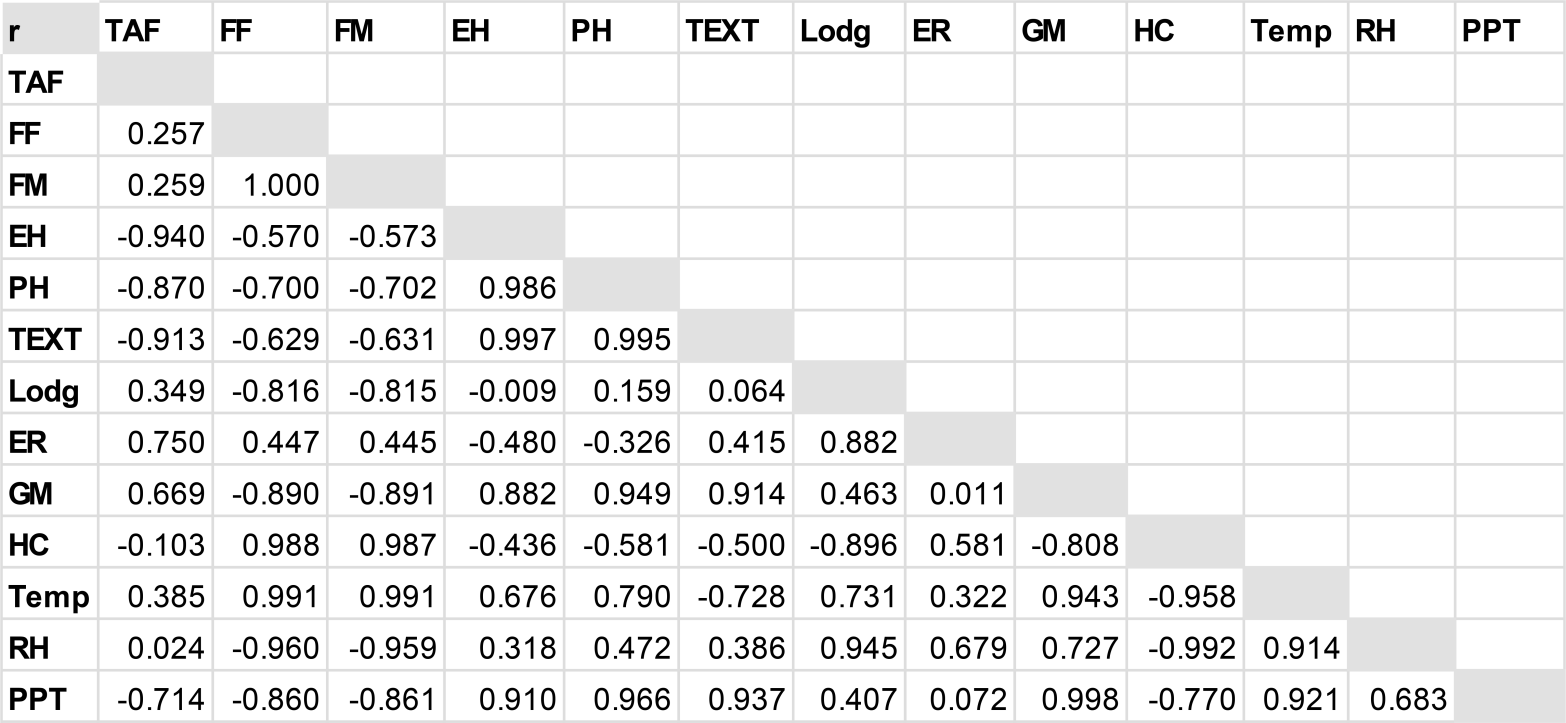
Correlation between traits/parameters associated with aflatoxin contamination in maize against aflatoxin contamination. TAF, Total Aflatoxin; FM, Male Flowering; FF, Female Flowering; PH, Plant Height; EH, Ear Height; Lodg, Lodging; FW, Field Weight (Unshelled cob/ear weight); GM, Grain Moisture; HC, Husk Cover; ER, Ear Rot; Text, Texture; r, correlation; Temp, Temperature; RH, Relative Humidity; PPT, Precipitation/Rainfall.

A pooled correlation matrix in **Figure 5** shows Total Aflatoxin has strong negative correlations with ear height (-0.940), plant height (-0.870), precipitation (-0.714), and kernel texture (-0.913), indicating that shorter plants, reduced rainfall, and softer kernels increase aflatoxin risk. Positive correlations with lodging (0349) and grain moisture (0.669) suggest that weaker stalks and higher kernel moisture promote contamination. Temperature (0.385) has a weak positive correlation, while relative humidity (0.024) has little effect. Weak correlations with flowering time (FF: 0.257, FM: 0.259) suggest minimal impact.

### 3.4 Principal component analysis for morphological variables associated with aflatoxin

#### 3.3.4.1 At Ilonga (Kilosa)

**Figure 6:**
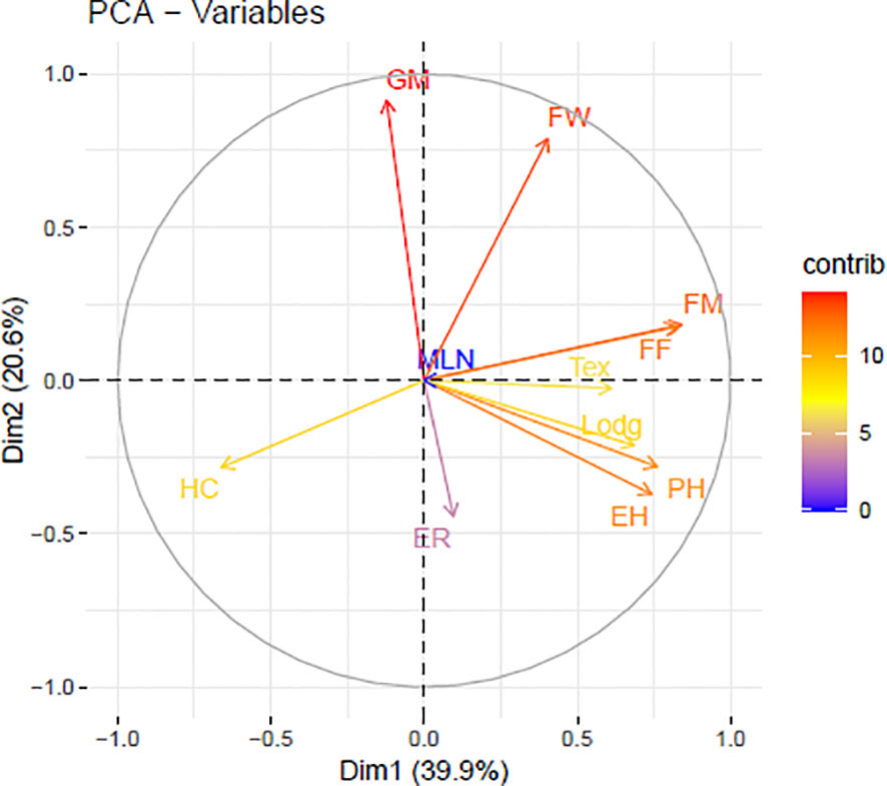
PCA biplot for variables associated with aflatoxin contamination at Ilonga. GM, Grain moisture; FW, Field weight (Unshelled cob/ear weight); FM, Male flowering (anthesis); FF, Female flowering (silking); Tex, Texture (kernel); Lodg, Lodging; PH, Plant height; EH, Ear height; ER, Ear rot; HC, Husk cover; contrib, Contribution; PCA, Principal Component Analysis.

The PCA biplot (**Figure 6**) accounts for 60.5% of the total variation, with Dim1 explaining 39.9% and Dim2 contributing 20.6%. Grain moisture (GM) and field weight (FW) are major contributors of variation, with GM being a key factor in aflatoxin contamination risk. GM was mainly associated with secondary variation, likely influenced by post-anthesis factors such as humidity and drying conditions. Flowering time (FM, FF), plant height (PH), and ear height (EH) primarily influence Dim1 and may indirectly affect susceptibility to aflatoxin. Husk cover (HC) is negatively correlated with plant height. Maize Lethal Necrosis (MLN) had weak associations with other traits.

#### 3.3.4.2 At SUA (Morogoro)

**Figure 7:**
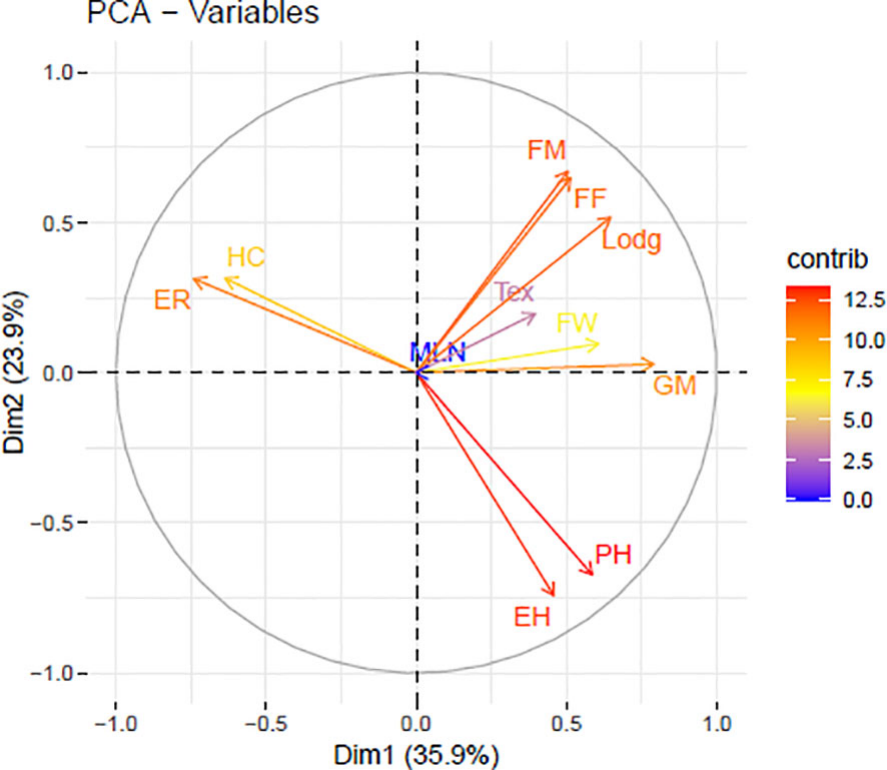
PCA biplot for variables associated with aflatoxin contamination at SUA.

The PCA biplot shows Dim1 (35.9%) and Dim2 (23.9%) explain most of the variation. Plant height (PH), ear height (EH), and flowering time (FM, FF) strongly contribute to Dim1, while ear rot (ER) and husk cover (HC) influence Dim2 (**Figure 7**). GM contributed more to the main trait structure, possibly due to more uniform conditions enhancing its association with yield traits.

#### 3.3.4.3 At Kirusix (Babati)

**Figure 8:**
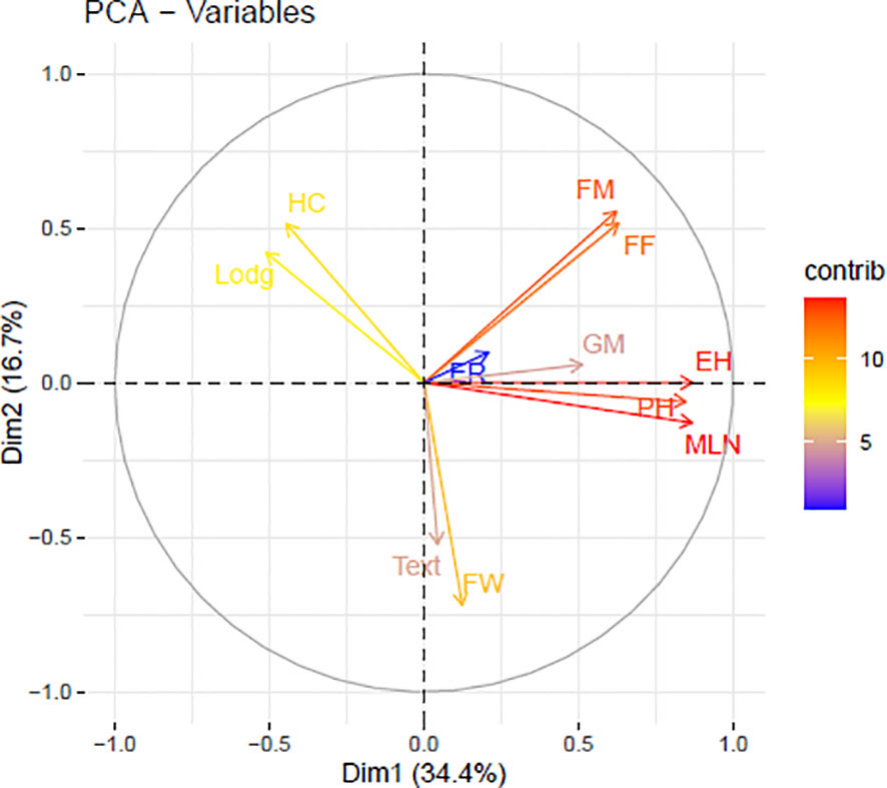
PCA biplot for variables associated with aflatoxin contamination at Kirusix.

The PCA biplot (**Figure 8**) shows Plant Height (PH), Ear Height (EH), and Male Flowering (FM) strongly contribute to Dim1 (34.4%), as indicated in red. Husk Cover (HC) and Lodging Resistance (Lodg) influence Dim2 (16.7%) but have a lesser impact. weaker correlations of GM, suggest greater environmental heterogeneity, differing harvest timing, or management effects that reduced consistency in trait relationships.

## 4 Discussion

### 4.1 Assessment of maize traits, variables and yield performance

#### 4.1.1 Assessment of agronomical, morphological, phenological traits in tested maize genotypes across the environments

The results (**Tables 3**-**5**) show that both environmental factors and genotypes significantly influence agronomic traits like plant height (PH), ear height (EH), and ear aspect (EA). The significant G × E interaction for several traits emphasises the need for multi-environment trials to identify stable genotypes, crucial for reducing Aspergillus infection and aflatoxin contamination. Netshifhefhe et al. (2018) and Rose et al. (2017) highlighted the importance of genotype by environmental analysis of Fusarium and fumonisin contamination. Aflatoxin accumulation is highly environment-dependent, influenced by factors such as temperature, humidity, and plant stress, so selecting resilient genotypes is essential. Genotypes with shorter plants, lower ear heights, and poor lodging resistance are more prone to soil fungal infection, highlighting the importance of moderate height and strong stalks to reduce exposure. Breeding programmes should focus on traits that reduce aflatoxin risk, particularly EH, PH, Text, and EA, which are genetically controllable.

#### 4.1.2 Assessment of foliar diseases in tested maize genotypes across the environments

MLN, MSV, and Rust severity were significantly influenced by the environment (**Table 6**), consistent with Kavai et al. (2025) and Nyaligwa (2014), while non-significant genotype (**Table 7**) and G × E effects (**Table 8**) indicate that foliar disease susceptibility was mainly environment-driven. However, similar disease responses do not imply equal vulnerability to *Aspergillus* infection or aflatoxin contamination, which are also shaped by physiological traits and environmental factors. Hussein (2023) noted that, conditions such as temperature, humidity, and plant stress can increase fungal growth and toxin production, emphasising the importance of environmental management in reducing disease and aflatoxin risks. Visual assessment alone cannot confirm susceptibility to Aspergillus flavus or aflatoxin contamination; therefore, Ultra-High Performance Liquid Chromatography (UHPLC) was used to confirm fungal presence and toxin occurrence.

#### 4.1.3 Assessment of *Aspergillus* ear rot and yield-related variables in tested maize genotypes

The study (**Table 9**) revealed significant variation in yield-related traits among maize genotypes, whereas genotypes A2207–4 and 2246 show high yield potential, with A2207–4 having the most ears and plants. Genotypes 5110 and 2246 also recorded the highest field weights. For ear rot resistance, 2743 and A2207–1 showed the lowest severity. These results highlight A2207–4 and 2246 as promising for breeding high-yield, disease-resistant maize. The findings suggest that selecting genotypes like A2207–4 and 2246, which have high yield and lower ear rot severity, can help develop productive and disease-resistant maize varieties. Breeding programmes should focus on these traits to improve maize productivity, emphasising the importance of balancing yield potential with disease resistance as supported by Horne et al. (2016); Lanubile et al. (2017) and Ndhlela (2012), as well as reflecting the challenges in addressing trade-offs in breeding programmes as reported by Nagaraja et al. (2024). The adaptability of certain genotypes across multiple environments suggests promising candidates for future breeding efforts.

#### 4.1.4 Assessment of AER and yield-related variables in tested environments

The results in **Table 10** indicate significant variation in yield-related traits across different sites. SUA achieved the highest yield, while Ilonga had low ear rot but also a lower yield, and Kirusix revealed moderate yields with high ear rot. These site-specific factors highlight the need for breeding strategies tailored to local conditions. Breeding programmes should focus on traits that optimise yield in areas like SUA and disease resistance in regions like Kirusix, ensuring maize varieties perform well across diverse environments. Sileshi et al. (2010) highlighted the role of soil type, climate, and nutrient inputs in yield gaps; moreover, Dalla Lana et al. (2021) indicated that increased humidity may have promoted fungal infections. The findings highlight the need for breeding site-specific maize genotypes with high yield potential and resistance to both biotic and abiotic stresses, as emphasised by Singh et al. (2024).

#### 4.1.5 Assessment of AER and yield-related variables in maize genotypes and their interaction with environments

Genotype × environment (G × E) interactions in **Table 12** significantly affected maize traits, especially ear rot (ER) and grain moisture (GM), with important implications for *Aspergillus* infection and aflatoxin contamination. Higher GM at SUA and Ilonga, combined with severe ER (up to 14% at SUA), created favourable conditions for fungal growth and mycotoxin production due to warm, humid environments. The previous studies by Mukanga et al. (2010) and Singamsetti et al. (2021) indicated the role of environmental factors such as temperature, precipitation and humidity in shaping crop performance. Zakaria (2024) reported that ear rot damages kernels, providing entry points for *Aspergillus* species, while Akumu et al. (2020); Kimatu et al. (2012), and Waliyar et al. (2015) indicated that moist grain increases post-harvest contamination risk. In contrast, Kirusix, with lower GM, may have reduced aflatoxin risk if proper drying and storage are practised. Although field weight (FW) remained stable across environments, breeding for genotypes with lower GM and ER resistance, alongside improved agronomic practices, is essential to minimise aflatoxin contamination and ensure safer maize production.

#### 4.1.6 Yield performance of tested maize genotypes across the environments

Significant yield variability was observed at SUA, reflecting greater environmental heterogeneity, while non-significant differences were found at Ilonga and Kirusix (**Table 13**). Environmental factors, such as humidity and temperature, are known to play a key role in ear rot prevalence, as reported by Njeru et al. (2020); Mukanga et al. (2010), and Price et al. (2024). Aflatoxin contamination in maize, caused by *Aspergillus* fungi, reduces both yield and quality. The fungi cause ear rot, which damages the kernels and disrupts the plant’s physiological processes through cell death, ultimately lowering productivity and grain quality. Given these constraints, Mahuku (2010) emphasises that host plant resistance is the most viable strategy for managing Aspergillus ear rot.

These findings highlight the need for breeding strategies that focus not only on high yield but also on disease resistance, particularly in environments prone to *Aspergillus* infection and aflatoxin contamination. As supported by Marenya et al. (2022) and Smith et al. (2001), selecting high-yielding genotypes remains a priority for farmers, but it must be balanced with resistance traits to optimise maize production and mitigate fungal damage.

### 4.2 Pre-harvest aflatoxin quantification in harvested maize grains

#### 4.2.1 Effects of genotypes on pre-harvest aflatoxin contamination of maize

Aflatoxin contamination varied significantly among maize genotypes, with A2207-4, 1, and AFL-Syn2-y showing lower levels (indicating resistance or tolerance to Aspergillus), while YANGA-5–2 and Genotype 12 were more susceptible (**Figure 3**; **Tables 15**, **16**). This demonstrates that, despite similar foliar disease responses, genotypes differ in aflatoxin resistance. These findings, supported by Ortega-Beltran and Cotty (2020), highlight the role of genetic resistance in limiting aflatoxin accumulation, while environmental factors such as temperature, humidity, and precipitation also influence contamination levels, as reported by Price et al. (2024). Overall, selecting low-aflatoxin genotypes and combining resistance with environmental adaptability is essential for improving grain quality and food safety.

#### 4.2.2 Effects of environments on pre-harvest aflatoxin contamination of maize

Ilonga (Kilosa) had the highest aflatoxin contamination compared to other locations, likely due to high humidity and low rainfall during the critical pre-harvest phase (flowering to harvesting) (**Figure 2**, **Table 16**). Payne (1998) indicated that low rainfall may weaken maize defences, causing kernel cracks that facilitate *Aspergillus* infection, while high humidity promotes fungal growth and increases pre-harvest aflatoxin contamination (Chalwe, 2020). Site-specific contamination differences emphasise the need for location-based management. Identifying stable, low-aflatoxin genotypes across environments is key to improving food safety and maize productivity. Furthermore, site-specific differences in contamination levels emphasise the need for location-based management strategies to mitigate aflatoxin risks effectively. Identifying stable genotypes with consistently low aflatoxin levels across environments is essential for improving food safety and maize productivity.

#### 4.2.3 Effects of genotype-by-environment interactions on pre-harvest aflatoxin contamination of maize

The significant genotype × environment (G × E) interaction in aflatoxin contamination (**Figure 4**, **Table 15**) indicates that both genetic resistance and environmental conditions influence *Aspergillus* infection. High contamination at Ilonga suggests that factors like high humidity and temperature as well as low rains between flowering and maturity could drive fungal growth. Some genotypes were highly susceptible, while others showed resistance, likely due to protective traits like husk cover and kernel texture. The highly significant effect emphasises the need for breeding resistant genotypes while implementing environmental management strategies to minimise aflatoxin risk, as reported by Ortega-Beltran and Bandyopadhyay (2021). Performance varied across sites, with some genotypes being more stable than others. The findings highlight the importance of selecting aflatoxin-resistant genotypes and considering environmental conditions to minimise contamination risks in maize production.

### 4.3 A pooled correlation between morphological traits/parameters associated with aflatoxin and aflatoxin contamination

This study (**Figure 5**) identifies key environmental and agronomic factors influencing aflatoxin contamination in maize. A negative correlation between total aflatoxin (TAF) and plant height (PH) and ear height (EH) suggests that shorter genotypes are more susceptible, likely due to proximity to soil-borne *Aspergillus* fungi (Munkvold and Desjardins, 1997); the relationship between shorter plants and high aflatoxin contamination was also reported by Pekar et al. (2019) and Wahl et al. (2017). Similarly, tight husks and flinty kernels provide protection against fungal invasion (Balconi et al., 2010; Mutiga et al., 2017).

Reduced rainfall results in the drought intensity which increases aflatoxin risk, as reported by Bhatnagar-Mathur et al. (2015). A strong positive correlation between TAF and *Aspergillus* ear rot (AER) confirms that ear rot susceptibility heightens aflatoxin accumulation risk; the same observation was reported by Henry et al. (2009). High grain moisture further promotes fungal growth, hence emphasising the need for proper grain drying (Liu et al., 2025). Lodging increases contamination risk by exposing plants to soil moisture and reducing airflow (Nakajima et al., 2014). While temperature slightly favours aflatoxin production, humidity has a weaker influence (Magan and Aldred, 2007). If the correlation is weak or insignificant, it implies that that trait/parameter alone is not a strong predictor of aflatoxin contamination, and other factors play a more significant role. These findings emphasise the importance of breeding taller, lodging-resistant maize genotypes, improving irrigation in drought-prone areas, and selecting varieties with good husk cover and ear rot resistance to mitigate aflatoxin contamination.

### 4.4 Principal component analysis for morphological variables associated with aflatoxin

#### 4.4.1 At Ilonga (Kilosa)

The PCA results (**Figure 6**) identify grain moisture (GM) as a key factor influencing aflatoxin contamination, supporting Muga et al. (2006), who linked high GM to fungal growth and aflatoxin contamination. Field weight (FW) also influences contamination, as Bandyopadhyay et al. (2016) noted that high field weight varieties with greater kernel moisture face increased aflatoxin risk. A positive correlation between FW and TAF suggests that heavier maize retains more moisture, promoting fungal proliferation. Conversely, Barošević et al. (2022) found that better lodging resistance and lower GM reduce aflatoxin susceptibility. Maize Lethal Necrosis (MLN) exhibits weak associations with other traits, with Mwasame (2021) also observing a slight positive correlation between TAF and MLN. Although MLN does not directly influence aflatoxin levels, MLN-induced stress may weaken plant defences, increasing susceptibility to secondary infections like Aspergillus fungi, as Gardisser et al. (2006) explained. These findings emphasise the need for integrated breeding strategies to enhance maize resilience, as highlighted by Lanubile et al. (2014) and Cary et al. (2011).

#### 4.4.2 At SUA (Morogoro)

The PCA results in **Figure 7** indicated that Plant Height (PH), Ear Height (EH), and Flowering Maturity (FM) have the highest contributions; the same findings were observed by Kushwah et al. (2024). Since PH, EH, and FM contribute significantly to genetic variation, breeders such as Yu et al. (2020) used PCA insights to select traits linked to lower aflatoxin contamination, such as optimal ear placement and moderate maturity periods. The negative correlation between GM and ER may be explained by biological and agronomic factors. Higher grain moisture at harvest can increase kernel susceptibility and prolong pathogen exposure, while faster-drying genotypes may reduce infection risk. In addition, delayed harvest, uneven field drying, and traits such as husk tightness and stress adaptation can modify ear microclimate and influence disease severity. The findings highlight the importance of integrating plant height, ear height, and flowering time into breeding programmes aimed at reducing aflatoxin contamination. Selecting genotypes with moderately tall plant heights, well-positioned ears, and moderate flowering maturity may be more resilient to aflatoxin accumulation.

#### 4.4.3 At Kirusix (Babati)

The PCA results highlight plant height (PH), ear height (EH), and flowering maturity (FM) as key traits influencing genetic variation and aflatoxin contamination risk (**Figure 8**). Optimal plant height and ear placement can reduce fungal infection, while moderate flowering maturity helps maize avoid drought stress, a major factor in aflatoxin accumulation, as reported by Munkvold (2014). The results suggest that selecting optimal PH, EH, and FM could be crucial in maize breeding programmes, particularly for traits linked to resilience against aflatoxin contamination and improved plant architecture.

## 5 Conclusion

Genotype × environment (G × E) interactions strongly influenced maize traits linked to Aspergillus infection and aflatoxin contamination, particularly ear rot and grain moisture. Sites with high moisture and disease severity (e.g., SUA and Ilonga) promoted fungal growth, highlighting the need for location-specific breeding.

Agronomic traits such as plant height, ear height, and flowering maturity affected aflatoxin levels, though inconsistently across environments. Resistant genotypes (A2207-4, AFL-Syn2-y) and promising landraces (1769, 2246) combined low aflatoxin with good yield, making them valuable for breeding. PCA and correlation analyses identified ear position, husk cover, and kernel texture as important in reducing fungal infection.

Overall, integrated breeding strategies are needed, and future studies should focus on fungal isolation and controlled manual inoculation to reliably assess genotype resistance.

## Data Availability

The original contributions presented in the study are included in the article/supplementary material. Further inquiries can be directed to the corresponding author.

## References

[B1] Abdallah M. F. Ameye M. De Saeger S. Audenaert K. Haesaert G. (2018). “ Biological control of mycotoxigenic fungi and their toxins: An update for the pre-harvest approach,” in Mycotoxins: Impact and management strategies (London, United Kingdom: IntechOpen). doi: 10.5772/intechopen.80452

[B2] Akumu G. Atukwase A. Tibagonzeka J. E. Apil J. Wambete J. M. Atekyereza P. R. . (2020). On-farm evaluation of effectiveness of improved postharvest handling of maize in reducing grain losses, mold infection and aflatoxin contamination in rural Uganda. Afr. J. Food Agric. Nutr. Dev. 20, 16522–16539. doi: 10.18697/ajfand.93.19790

[B3] AOAC (2006). AOAC official method 2005.08 Aflatoxins in corn, raw peanuts, and peanut butter. Liquid chromatography with post-column photochemical derivatisation. J. AOAC Int. 89, 678–679. doi: 10.1093/jaoac/89.3.678 16792067

[B4] Balconi C. Motto M. Mazzinelli G. Berardo N. (2010). Ear secondary traits related to aflatoxin accumulation in commercial maize hybrids under artificial field inoculation. World Mycotoxin J. 3, 239–250. doi: 10.3920/wmj2010.1205

[B5] Bandyopadhyay R. Ortega-Beltran A. Akande A. Mutegi C. Atehnkeng J. Kaptoge L. . (2016). Biological control of aflatoxins in Africa: current status and potential challenges in the face of climate change. World Mycotoxin J. 9, 771–790. doi: 10.3920/wmj2016.2130

[B6] Barošević T. Bagi F. Savić Z. Ljubičić N. Ivanović I. (2022). Assessment of maize hybrids resistance to Aspergillus ear rot and aflatoxin production in environmental conditions in Serbia. Toxins 14, 887. doi: 10.3390/toxins14120887 36548784 PMC9781229

[B7] Bello O. B. Abdulmaliq S. Y. Ige S. Mahamood J. Oluleye F. Azeez M. A. . (2012). Evaluation of early and late/intermediate maize varieties for grain yield potential and adaptation to a southern Guinea savanna agro-ecology of Nigeria. Int. J. Plant Res. 2, 14–21. doi: 10.5923/j.plant.20120202.03

[B8] Benkerroum N. (2020). Aflatoxins: Producing moulds, structure, health issues, and incidence in Southeast Asian and Sub-Saharan African countries. Int. J. Environ. Res. Public Health 17, 1215. doi: 10.20944/preprints201911.0350.v3 32070028 PMC7068566

[B9] Benvenuti M. E. Di Gioia A. J. (2009). “ Rapid analysis of aflatoxins without derivatisation using ultra-performance liquid chromatography and fluorescence detection,” in Waters application note. (Milford, MA, USA: Waters Corporation).

[B10] Bhatnagar-Mathur P. Sunkara S. Bhatnagar-Panwar M. Waliyar F. Sharma K. K. (2015). Biotechnological advances for combating Aspergillus flavus and aflatoxin contamination in crops. Plant Sci. 234, 119–132. doi: 10.1016/j.plantsci.2015.02.009. PMID: 25804815

[B11] Bilal E. K. Owaga E. E. Njoroge D. M. (2023). Occurrence of aflatoxigenic fungi and aflatoxins in maize grains and associated awareness and handling practices among farmers and traders in South Sudan. Afr. J. Food Agric. Nutr. Dev. 23, 24801–24824. doi: 10.18697/ajfand.125.23920

[B12] Boni S. B. Beed F. Kimanya M. E. Koyano E. Mponda O. Mamiro D. . (2021). Aflatoxin contamination in Tanzania: quantifying the problem in maize and groundnuts from rural households. World Mycotoxin J. 14, 553–564. doi: 10.3920/wmj2020.2646

[B13] Cai Y. McLaughlin M. Zhang K. (2020). Advancing the FDA/Office of Regulatory Affairs mycotoxin program: new analytical method approaches to addressing needs and challenges. J. AOAC Int. 103, 705–709. doi: 10.1093/jaocint/qsz007. PMID: 33241365

[B14] Caldu-Primo J. L. Mastretta-Yanes A. Wegier A. Piñero D. (2017). Finding a needle in a haystack: distinguishing Mexican maize landraces using a small number of SNPs. Front. Genet. 8, 45. doi: 10.3389/fgene.2017.00045. PMID: 28458682 PMC5394175

[B15] Cary J. W. Rajasekaran K. Brown R. L. Luo M. Chen Z. Y. Bhatnagar D. (2011). Developing resistance to aflatoxin in maize and cottonseed. Toxins 3, 678–696. doi: 10.3390/toxins3060678. PMID: 22069734 PMC3202838

[B16] Chalwe H. M. (2020). Modeling pre-harvest aflatoxin incidence in groundnut (arachis hypogaea l.) using selected soil properties and ambient temperature. (Master’s thesis). (Lusaka, Zambia: The University of Zambia).

[B17] Dalla Lana F. Madden L. V. Paul P. A. (2021). Natural occurrence of maize Gibberella ear rot and contamination of grain with mycotoxins in association with weather variables. Plant Dis. 105, 114–126. doi: 10.1094/pdis-05-20-0952-re. PMID: 33197383

[B18] Doose S. Leslie J. F. Bandyopadhyay R . (2019). Biological control of aflatoxins in maize: Current status and future prospects. Toxins 11 (10), 595. doi: 10.3390/toxins11100595 31614800 PMC6832162

[B19] Dwivedi S. L. Ceccarelli S. Blair M. W. Upadhyaya H. D. Are A. K. Ortiz R. (2016). Landrace germplasm for improving yield and abiotic stress adaptation. Trends Plant Sci. 21, 31–42. doi: 10.1016/j.tplants.2015.10.012. PMID: 26559599

[B20] East African Community (2011). EAS 2:2011 maize grains—Specification (Arusha, Tanzania: East African Standards Committee).

[B21] Eskola M. Kos G. Elliott C. T. Hajšlová J. Mayar S. Krska R. (2020). Worldwide contamination of food crops with mycotoxins: Validity of the widely cited ‘FAO estimate of 25%. Crit. Rev. Food Sci. Nutr. 60, 2773–2789. doi: 10.1080/10408398.2019.1658570 31478403

[B22] European Commission . (2010). Commission Regulation (EU) No 165/2010 of 26 February 2010 amending Regulation (EC) No 1881/2006 setting maximum levels for certain contaminants in foodstuffs as regards aflatoxins (Belgium: European Commission).

[B23] Food and Agriculture Organization of the United Nations . (2018). Manual on sampling and analysis of aflatoxins in food and feed (Rome, Italy: FAO).

[B24] Food Business MEA . (2021). Kenya raises concerns on safety of maize imported from Tanzania and Uganda. Available online at: https://www.foodbusinessmea.com/Kenya-raises-concerns-on-safety-of-maize-imported-from-tanzania-uganda (Accessed May 26, 2026).

[B25] Frisvad J. C. Hubka V. Ezekiel C. N. Hong S. B. Novßkovß A. Chen A. J. . (2019). Taxonomy of Aspergillus section Flavi and their production of aflatoxins, ochratoxins, and other mycotoxins. Stud. Mycol. 93, 1–63. doi: 10.3114/sim.2011.70.02. PMID: 30108412 PMC6080641

[B26] Gardisser D. Huitink G. Cartwright R. (2006). “ 10-grain storage and aflatoxin in corn,” in Corn production handbook, (Little Rock, AR, USA: University of Arkansas Division of Agriculture, Cooperative Extension Service), 79–85.

[B27] Henry W. B. Williams W. P. Windham G. L. Hawkins L. K. (2009). Evaluation of maize inbred lines for resistance to Aspergillus and Fusarium ear rot and mycotoxin accumulation. Agron. J. 101, 1219–1226. doi: 10.2134/agronj2009.0004

[B28] Herrman T. J. Lee K. M. Jones B. McCormick C. (2014). Aflatoxin sampling and testing proficiency in the Texas grain industry. J. Regul. Sci. 2, 7–13. doi: 10.21423/jrs.regsci.2112

[B29] Horne D. W. Eller M. S. Holland J. B. (2016). Responses to recurrent index selection for reduced Fusarium ear rot and lodging and for increased yield in maize. Crop Sci. 56, 85–94. doi: 10.2135/cropsci2015.06.0333

[B30] Hussein M. A. (2023). Maize post-harvest handling, knowledge and practices of farmers in Somalia (a case of maize meal aflatoxin exposure). (Master’s thesis). (Nairobi, Kenya: University of Nairobi).

[B31] Joutsjoki V. V. Korhonen H. J. (2021). Management strategies for aflatoxin risk mitigation in maize, dairy feeds, and milk value chains—case study Kenya. Food Qual. Saf. 5, fyab005. doi: 10.1093/fqsafe/fyab005. PMID: 40388063

[B32] Kamala A. Kimanya M. Haesaert G. Tiisekwa B. Madege R. Degraeve S. . (2016). Local post-harvest practices associated with aflatoxin and fumonisin contamination of maize in three agro-ecological zones of Tanzania. Food Addit. Contam. 33, 551–559. doi: 10.1080/19440049.2016.1138546. PMID: 26795400

[B33] Kamala A. Shirima C. Jani B. Bakari M. Sillo H. Rusibamayila N. . (2018). Outbreak of an acute aflatoxicosis in Tanzania during 2016. World Mycotoxin J. 11, 311–320. doi: 10.3920/WMJ2018.2344

[B34] Kavai H. M. Makumbi D. Nzuve F. M. Woyengo V. W. Suresh L. M. Muiru W. M. . (2025). Inheritance of resistance to maize lethal necrosis in tropical maize inbred lines. Front. Plant Sci. 15, 1506139. doi: 10.3389/fpls.2024.1506139. PMID: 39850213 PMC11753913

[B35] Kebede H. Liu X. Jin J. Xing F. (2020). Current status of major mycotoxins contamination in food and feed in Africa. Food Control 110, 106975. doi: 10.1016/j.foodcont.2019.106975. PMID: 38826717

[B36] Kimatu J. N. McConchie R. Xie X. Nguluu S. N. (2012). The significant role of post-harvest management in farm management, aflatoxin mitigation and food security in Sub-Saharan Africa. Greener J. Agric. Sci. 2, 279–288.

[B37] Kovalsky P. Kos G. Nährer K. Schwab C. Jenkins T. Schatzmayr G. . (2016). Co-occurrence of regulated, masked and emerging mycotoxins and secondary metabolites in finished feed and maize—An extensive survey. Toxins, 8 (12), 363. doi: 10.3390/toxins8120363 27929415 PMC5198557

[B38] Kushwah A. Yousuf N. Sandhu S. Singh G. Garg T. Ranjan R. . (2024). Heterotic analysis and yield assessment of maize (Zea mays L.) inbred lines for developing superior hybrids under sub-tropical climatic conditions. Vegetos 37 (5), 1948–1955. doi: 10.1007/s42535-023-00796-x. PMID: 30311153

[B39] Lanubile A. Maschietto V. Borrelli V. M. Stagnati L. Logrieco A. F. Marocco A. (2017). Molecular basis of resistance to Fusarium ear rot in maize. Front. Plant Sci. 8, 1774. doi: 10.3389/fpls.2017.01774. PMID: 29075283 PMC5644281

[B40] Lanubile A. Maschietto V. Marocco A. (2014). “ Breeding maize for resistance to mycotoxins,” in Mycotoxin reduction in grain chains, eds. Leslie J. F. Logrieco A. ( John Wiley & Sons), 37–58.

[B41] Liu C. Chen G. Zheng D. Yin J. Cui C. Lu H. (2025). Analysis of heat and moisture transfer and fungi-induced hot spots in maize bulk with different broken kernel contents. Agriculture 15, 338. doi: 10.3390/agriculture15030338. PMID: 30654563

[B42] Logrieco A. F. Miller J. D. Eskola M. Krska R. Ayalew A. Bandyopadhyay R. . (2018). The mycotoxin charter: increasing awareness of, and concerted action for minimising, mycotoxin exposure worldwide. Toxins (Basel) 10, 149. doi: 10.3390/toxins10040149. PMID: 29617309 PMC5923315

[B43] Magan N. Aldred D. (2007). Post-harvest control strategies: minimizing mycotoxins in the food chain. Int. J. Food Microbiol. 119, 131–139. doi: 10.1016/j.ijfoodmicro.2007.07.034. PMID: 17764773

[B44] Magembe K. S. Mwatawala M. W. Mamiro D. P. Chingonikaya E. E. (2016). Assessment of awareness of mycotoxin infections in stored maize (Zea mays L.) and groundnut (Arachis hypogea L.) in Kilosa District, Tanzania. Int. J. Food Contam. 3, 1–8. doi: 10.1186/s40550-016-0035-5

[B45] Mahuku G. (2010). “ Maize pathology in Asia: opportunities and challenges for breeding disease-resistant maize,” in Proceedings of the Asian Regional Maize Workshop. (Bangkok, Thailand: CIMMYT) vol. 10, 361–366.

[B46] Mahuku G. Nzioki H. S. Mutegi C. Kanampiu F. Narrod C. Makumbi D. (2019). Pre-harvest management is a critical practice for minimizing aflatoxin contamination of maize. Food Control 96, 219–226. doi: 10.1016/j.foodcont.2018.08.032. PMID: 30713368 PMC6251936

[B47] Mannaa M. Kim K. D. (2017). Influence of temperature and water activity on deleterious fungi and mycotoxin production during grain storage. Mycobiology 45, 240–254. doi: 10.5941/myco.2017.45.4.240. PMID: 29371792 PMC5780356

[B48] Massomo S. M. S . (2020). Aspergillus flavus and aflatoxin contamination in the maize value chain and what needs to be done in Tanzania. Scientific African, 10, e00606. doi: 10.1016/j.sciaf.2020.e00606

[B49] Marenya P. Wanyama R. Alemu S. Westengen O. Jaleta M. (2022). Maize variety preferences among smallholder farmers in Ethiopia: Implications for demand-led breeding and seed sector development. PloS One 17, e0274262. doi: 10.1371/journal.pone.0274262. PMID: 36174004 PMC9522265

[B50] Muga F. C. Marenya M. O. Workneh T. S . (2006). Effect of temperature, relative humidity and moisture on aflatoxin contamination of stored maize kernels. Bulg. J. Agric. Sci. 25, 271–277.

[B51] Mukanga M. Derera J. Tongoona P. Laing M. D. (2010). A survey of pre-harvest ear rot diseases of maize and associated mycotoxins in south and central Zambia. Int. J. Food Microbiol. 141, 213–221. doi: 10.1016/j.ijfoodmicro.2010.05.011. PMID: 20626099

[B52] Munkvold G. (2014). “ Crop management practices to minimize the risk of mycotoxins contamination in temperate‐zone maize,” in Mycotoxin reduction in grain chains, eds. Leslie J. F. Logrieco A. (Ames, IA, USA: Wiley-Blackwell), 59–77.

[B53] Munkvold G. P. Desjardins A. E. (1997). Fumonisins in maize: can we reduce their occurrence? Plant Dis. 81, 556–565. doi: 10.1094/pdis.1997.81.6.556. PMID: 30861834

[B54] Mutiga S. K. Morales L. Angwenyi S. Wainaina J. Harvey J. Das B. . (2017). Association between agronomic traits and aflatoxin accumulation in diverse maize lines grown under two soil nitrogen levels in Eastern Kenya. Field Crops Res. 205, 124–134. doi: 10.1016/j.fcr.2017.02.007. PMID: 38826717

[B55] Mwasame E. N. (2021). Correlation between maize lethal necrosis disease and mycotoxin in maize in bomet, narok and nakuru counties, Kenya. (Master’s thesis). (Nairobi, Kenya: Kenyatta University).

[B56] Nagaraja T. E. Parveen S. G. Aruna C. Hariprasanna K. Singh S. P. Singh A. K. . (2024). Millets and pseudocereals: A treasure for climate-resilient agriculture ensuring food and nutrition security. Indian J. Genet. Plant Breed. 84, 1–37. doi: 10.31742/isgpb.84.1.1

[B57] Nakajima M. Proctor R. H. Duran R. M. Horiuchi H. Takanashi K. Kistler H. C. . (2014). Functional analysis of the aflatoxin biosynthetic gene cluster in Aspergillus flavus. Fungal Genet. Biol. 73, 1–12. doi: 10.1016/j.fgb.2014.08.001 25234739

[B58] Ndhlela T. (2012). Improvement strategies for yield potential, disease resistance, and drought tolerance of Zimbabwean maize inbred lines. (Doctoral dissertation). (Bloemfontein, South Africa: University of the Free State).

[B59] Netshifhefhe N. E. I. Flett B. C. Viljoen A. Rose L. J. (2018). Inheritance and genotype by environment analyses of resistance to Fusarium verticillioides and fumonisin contamination in maize F 1 hybrids. Euphytica 214, 1–20. doi: 10.1007/s10681-018-2310-4. PMID: 30311153

[B60] Niaz W. Iqbal S. Z. Ahmad K. Majid A. Haider W. Li X. (2025). Mycotoxins: A comprehensive review of its global trends in major cereals, advancements in chromatographic detections and future prospectives. Food. Chemistry: X 27, 102350. doi: 10.1016/j.fochx.2025.102350. PMID: 40213337 PMC11984607

[B61] Njeru N. K. Midega C. A. Muthomi J. W. Wagacha J. M. Khan Z. R. (2020). Impact of push–pull cropping system on pest management and occurrence of ear rots and mycotoxin contamination of maize in western Kenya. Plant Pathol. 69, 1644–1654. doi: 10.1111/ppa.13259. PMID: 40046247

[B62] Nyaligwa L. M. (2014). Genetic analysis, combining ability and yield stability of maize genotypes under maize streak virus prone environments. (Doctoral dissertation). (Pietermaritzburg: University of KwaZulu-Natal).

[B63] Nyangi C. (2016). Aflatoxin and fumonisin contamination of at-harvest and storage beans in Babati district, Northern Tanzania. Greener J. Agric. Sci. 6, 304–310. doi: 10.15580/gjas.2016.10.101916186

[B64] Odindo B. A. (2017). Genetic analysis of resistance to aspergillus ear rot and aflatoxin accumulation in maize (Zea mays L.) inbred lines. (Master’s thesis). (Nairobi, Kenya: University of Nairobi).

[B65] Okoth S. Nyongesa B. Ayugi V. Kang’ethe E. K. Korhonen H. Joutsjoki V. (2009). Toxigenic potential of Aspergillus flavus and Aspergillus parasiticus in maize kernels from two agro-ecological zones in Kenya. Toxins 1 (3), 106–123. doi: 10.3390/toxins1030106 PMC3509695 23202303

[B66] Oluwarant A. E. Adebowale A. A . (2015). Effects of storage conditions on aflatoxin contamination of maize grains. Afr. J. Food Sci. 9 (5), 270–277. doi: 10.5897/AJFS2015.1328

[B67] Ortega-Beltran A. Bandyopadhyay R. (2021). Contributions of integrated aflatoxin management strategies to achieve the sustainable development goals in various African countries. Global Food Secur. 30, 100559. doi: 10.1016/j.gfs.2021.100559. PMID: 38826717

[B68] Ortega-Beltran A. Cotty P. J. (2020). Influence of wounding and temperature on resistance of maize landraces from Mexico to aflatoxin contamination. Front. Plant Sci. 11, 572264. doi: 10.3389/fpls.2020.572264. PMID: 33072148 PMC7541827

[B69] Payne G. A. (1998). Process of contamination by aflatoxin-producing fungi and their impact on crops. Mycotoxins Agric. Food. Saf. 9, 279–306. doi: 10.1201/9780203752648-11

[B70] Pekar J. J. Murray S. C. Isakeit T. S. Scully B. T. Guo B. Knoll J. E. . (2019). Evaluation of elite maize inbred lines for reduced Aspergillus flavus infection, aflatoxin accumulation, and agronomic traits. Crop Sci. 59, 2562–2571. doi: 10.2135/cropsci2019.04.0206

[B71] Pérez-Escamilla R. (2017). Food security and the 2015–2030 sustainable development goals: From human to planetary health. Curr. Dev. Nutr. 1, e000513. doi: 10.1016/b978-0-12-821848-8.00067-6. PMID: 29955711 PMC5998358

[B72] Ponce-García N. Palacios-Rojas N. Serna-Saldivar S. O. García-Lara S. (2021). Aflatoxin contamination in maize: occurrence and health implications in Latin America. World Mycotoxin J. 14, 247–258. doi: 10.3920/wmj2020.2666

[B73] Price J. L. Visagie C. M. Meyer H. Yilmaz N. . (2024). Fungal species and mycotoxins associated with maize ear rots collected from the Eastern Cape in South Africa. Toxins 16 (2), 95. doi: 10.3390/toxins16020095 38393173 PMC10891880

[B74] R Core Team (2024). R: A language and environment for statistical computing (Version 4.4.2) (Computer software), (Vienna, Austria: R Foundation for Statistical Computing). Available online at: https://www.R-project.org (Accessed May 24, 2026).

[B75] Richard J. L . (2007). Some major mycotoxins and their mycotoxicoses—An overview. Int. J. Food Microbiol. 119 (1–2), 3–10. doi: 10.1016/j.ijfoodmicro.2007.07.019 17719115

[B76] Romero Navarro J. A. Willcox M. Burgueño J. Romay C. Swarts K. Trachsel S. . (2017). A study of allelic diversity underlying flowering-time adaptation in maize landraces. Nat. Genet. 49, 476–480. doi: 10.1038/ng.3784. PMID: 28166212

[B77] Rose L. J. Okoth S. Beukes I. Ouko A. Mouton M. Flett B. C. . (2017). Determining resistance to Fusarium verticillioides and fumonisin accumulation in African maize inbred lines resistant to Aspergillus flavus and aflatoxins. Euphytica 213, 1–18. doi: 10.1007/s10681-017-1883-7. PMID: 30311153

[B78] Shabeer S. Asad S. Jamal A. Ali A. (2022). Aflatoxin contamination, its impact, and management strategies: an updated review. Toxins 14, 307. doi: 10.3390/toxins14050307. PMID: 35622554 PMC9147583

[B79] Sileshi G. Akinnifesi F. K. Debusho L. K. Beedy T. Ajayi O. C. Mong’omba S. (2010). Variation in maize yield gaps with plant nutrient inputs, soil type, and climate across sub-Saharan Africa. Field Crops Res. 116, 1–13. doi: 10.1016/j.fcr.2009.11.014. PMID: 38826717

[B80] Singamsetti A. Shahi J. P. Zaidi P. H. Seetharam K. Vinayan M. T. Kumar M. . (2021). Genotype × environment interaction and selection of maize (Zea mays L.) hybrids across moisture regimes. Field Crops Res. 270, 108224. doi: 10.1016/j.fcr.2021.108224. PMID: 38826717

[B81] Singh S. B. Kumar B. Singh A. Kumar S. (2024). “ Conventional and advanced breeding approaches for developing abiotic stress-tolerant maize,” in Adapting to climate change in agriculture—Theories and practices: approaches for adapting to climate change in agriculture in India ( Springer Nature Switzerland, Cham), 281–302.

[B82] Smith M. E. Castillo F. G. Gómez F. (2001). Participatory plant breeding with maize in Mexico and Honduras. Euphytica 122, 551–563. doi: 10.1023/a:1017510529440. PMID: 41886696

[B83] Solaimalai A. Anantharaju P. Irulandi S. Theradimani M. (2020). Maize crop: improvement, production, protection, and post-harvest technology. (Boca Raton, FL, USA: CRC Press (Taylor & Francis Group)).

[B84] Streit E. Schwab C. Sulyok M. Naehrer K. Krska R. Schatzmayr G. (2013). Multi-mycotoxin screening reveals the occurrence of 139 different secondary metabolites in feed and feed ingredients. Toxins 5 (3), 504–523. doi: 10.3390/toxins5030504 23529186 PMC3705275

[B85] Tanzania Meteorological Authority . (2024). Statement on the status of Tanzania climate in 2023. Available online at: https://www.meteo.go.tz (Accessed 2024).

[B86] The Citizen . (2021). Kenya bans maize imports from Tanzania and Uganda. Available online at: https://www.thecitizen.co.tz/Tanzania/news/national/Kenya-bans-maize-imports-from-Tanzania-and-Uganda-bashe-calls-for-calm-3313910 (Accessed May 24, 2026).

[B87] Udomkun P. Wiredu A. N. Nagle M. Bandyopadhyay R. Müller J. Vanlauwe B. (2017). Mycotoxins in Sub-Saharan Africa: Present situation, socio-economic impact, awareness, and outlook. Food Control 72, 110–122. doi: 10.1016/j.foodcont.2016.07.039. PMID: 38826717

[B88] Wahl N. Murray S. C. Isakeit T. Krakowsky M. Windham G. L. Williams W. P. . (2017). Identification of resistance to aflatoxin accumulation and yield potential in maize hybrids in the Southeast Regional Aflatoxin Trials (SERAT). Crop Sci. 57, 202–215. doi: 10.2135/cropsci2016.06.0519

[B89] Waliyar F. Osiru M. Ntare B. R. Kumar K. V. K. Sudini H. Traore A. . (2015). Post-harvest management of aflatoxin contamination in groundnut. World Mycotoxin J. 8, 245–252. doi: 10.3920/wmj2014.1766

[B90] Warburton M. L. Rauf S. Marek L. Hussain M. Ogunola O. de Jesus Sanchez Gonzalez J. (2017). The use of crop wild relatives in maize and sunflower breeding. Crop Sci. 57, 1227–1240. doi: 10.2135/cropsci2016.10.0855

[B91] White J. F . (2016). “ Mycotoxins in agriculture and food safety: An overview.” in Mycotoxins in agriculture and food safety. ( CRC Press), 1–20. doi: 10.1201/9781315370733

[B92] Yu B. Jiang H. Pandey M. K. Huang L. Huai D. Zhou X. . (2020). Identification of two novel peanut genotypes resistant to aflatoxin production and their SNP markers associated with resistance. Toxins 12, 156. doi: 10.3390/toxins12030156. PMID: 32121605 PMC7150746

[B93] Zakaria L. (2024). An overview of Aspergillus species associated with plant diseases. Pathogens 13, 813. doi: 10.3390/pathogens13090813. PMID: 39339004 PMC11435247

